# The three causal pathways of ENSO teleconnections to High Mountain Asia winter precipitation

**DOI:** 10.1007/s00382-026-08198-w

**Published:** 2026-06-01

**Authors:** Pritam Jyoti Borah, Antonios Mamalakis, Clement Guilloteau, Alejandro Tejedor, Efi Foufoula-Georgiou

**Affiliations:** 1 Department of Civil and Environmental Engineering, University of California Irvine, Irvine, CA USA; 2 Department of Earth System Science, University of California Irvine, Irvine, CA USA; 3 Department of Environmental Sciences, University of Virginia, Charlottesville, VA USA; 4 School of Data Science, University of Virginia, Charlottesville, VA USA; 5 Institute for Biocomputation and Physics of Complex Systems (BIFI), University of Zaragoza, Zaragoza, Spain; 6 Department of Theoretical Physics, University of Zaragoza, Zaragoza, Spain

**Keywords:** ENSO teleconnections, High Mountain Asia, Winter precipitation, Causal discovery

## Abstract

High Mountain Asia (HMA), spanning from the Hindu Kush to the Tibetan Plateau, encompasses tropical and subtropical regions highly susceptible to extreme precipitation events and associated hazards. El Niño–Southern Oscillation (ENSO) is one of the dominant external climate modes that influence subseasonal to seasonal precipitation over HMA through various dynamical pathways. We hypothesize three possible ENSO-driven teleconnection pathways impacting HMA precipitation and test their causality using a data-driven causal discovery method, PCMCI+ , an improved version of the Peter and Clark Momentary Conditional Independence algorithm. The three physically reasoned ENSO- driven teleconnection pathways are (1) extratropical Rossby wave response (EWP), (2) tropical moisture transport from the Indian ocean (TMP) and (3) the subtropical westerly jet modulation (SJP) towards HMA. Contrary to most prior studies that rely on simple correlation analysis to establish ENSO-HMA precipitation relationships, PCMCI + offers a rigorous causal discovery method for high dimensional interdependent time series, based on graphical causal models for establishing causal links and estimating their strength. Our analysis shows that HMA November precipitation is modulated by an ENSO extratropical Rossby wave teleconnection and by tropical moisture transport (EWP and TMP), while March precipitation is influenced through the subtropical jet (SJP). Moreover, by quantifying the causal effect of ENSO with robust causal network guided regression, we establish how a change on ENSO would propagate to variations in HMA precipitation. These findings offer critical insights for improving winter precipitation forecasts over HMA, diagnosing physics-based models, and examining future changes under internal and forced climate variability.

**Supplementary Information:**

The online version contains supplementary material available at 10.1007/s00382-026-08198-w.

## Plain language summary

High Mountain Asia (HMA) in the Hindu-Kush and western Himalayas receives large amounts of precipitation in winter making it highly susceptible to floods and land hazards. El Niño Southern Oscillation (ENSO) is one of the dominant climate modes known to influence HMA winter precipitation, yet the causal pathways of this influence remain speculative so far. In this study we used a graphical causal network framework to examine how ENSO modulates intermediate atmospheric patterns affecting HMA winter precipitation. We tested three physically hypothesized ENSO driven pathways, i.e. midlatitude waves, subtropical jet stream and tropical moisture transport to bring out ENSO’s possible influence on HMA precipitation. Our causal discovery analysis shows that ENSO influences the November precipitation through the combined role of midlatitude waves and local moisture transport, while March precipitation is influenced by modulation of the subtropical jet strength.

**Supplementary Information:**

The online version contains supplementary material available at 10.1007/s00382-026-08198-w.

## Introduction

**Fig. 1:**
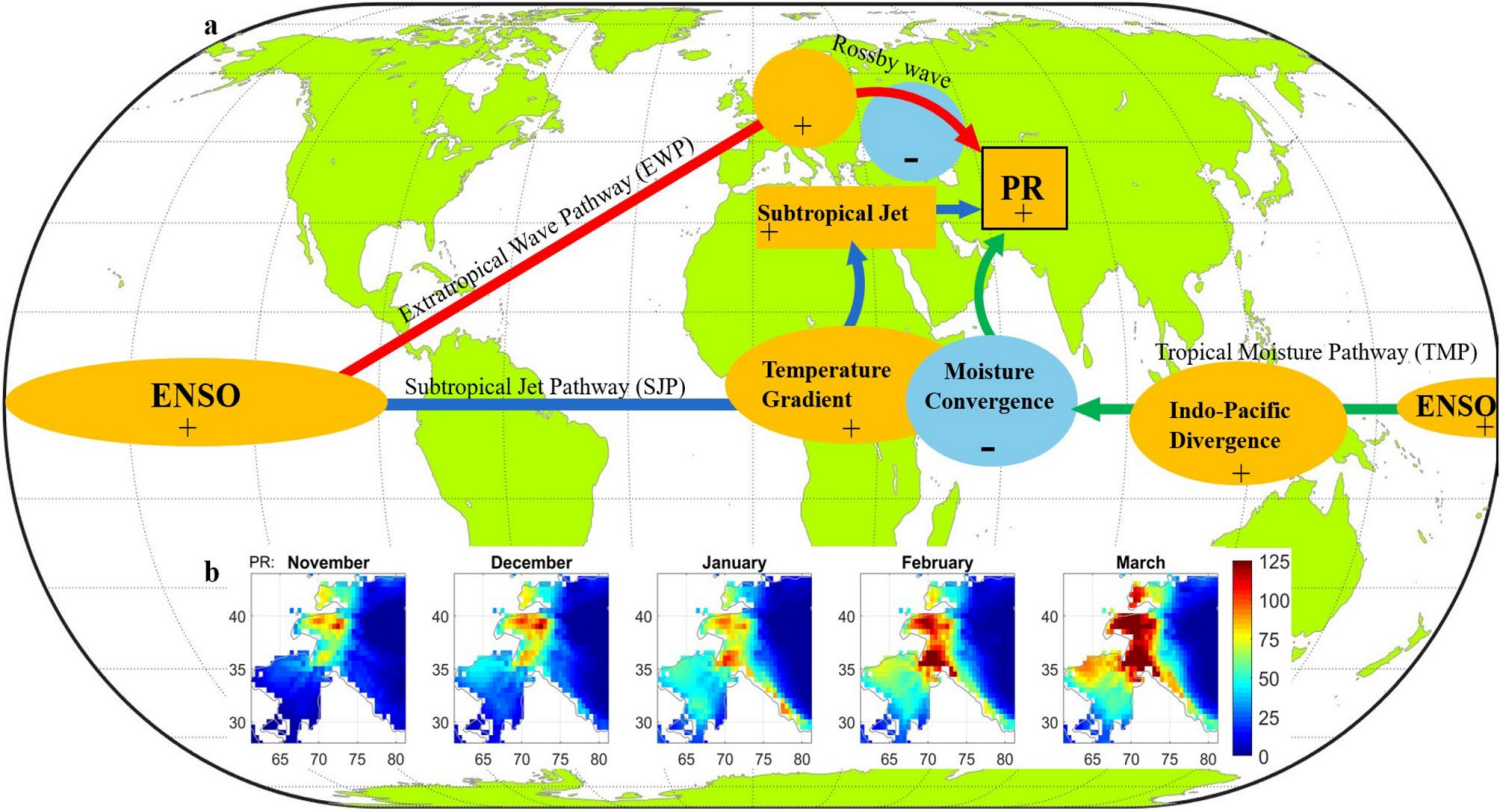
**a** The three hypothesized ENSO teleconnection pathways influencing HMA winter precipitation: Extratropical Wave Pathway (EWP), Subtropical Jet Pathway (SJP), and Tropical Moisture Pathway (TMP). The observables used to represent the oceanic and atmospheric processes involved in the 3 pathways are listed as follows, ENSO as sea surface temperature (SST), Extratropical wave as geopotential height at 200 hPa (Z200), temperature gradient as Lower atmospheric air temperature (500-700 hPa), Subtropical westerly jet as zonal wind at 200 hPa (U200), Indo-Pacific lower level divergence as geopotential height at 850 hPa (Z850), and moisture convergence as vertically integrated moisture divergence (VIMD), **b** Climatological HMA winter precipitation (mm) during November-March (UK-CRU data)

The High Mountain Asia (HMA) is generally considered as the Asian mountain regions (Fig. S1) with geographic elevation above 1 km (Nash et al. 2022; Roy and Singh; 2024). It encompasses landmasses of nine Asian countries from Hindu-Kush Mountains to eastern Himalayas (Maina et al. 2022), consisting of multiple hydrological basins, glaciers and lakes with heterogeneous hydroclimate (Song et al. 2016; Yoon et al. 2019), on which hundreds of millions of people depend. HMA regularly experiences extreme precipitation and associated hydrometeorological hazards (Kirschbaum et al. 2020; Nash et al. 2024). While spatio-temporal variability of HMA precipitation (Fig. 1b) is largely guided by regional orography and local circulation patterns, regional climate and remote external forcings also exert influence. The majority of winter precipitation (rainfall + snowfall) in HMA (~ 310 mm) is observed over the Hindu-Kuch Mountain and part of western Himalayas (Fig. 1b), which is the focus area of our study.

The wintertime precipitation in HMA is caused by cyclonic storms (or western disturbances) travelling along the subtropical jet stream from the Mediterranean region (Cannon et al. 2015; Nash et al. 2022; Lyngwa et al. 2023; Hunt et al. 2025), the influence of midlatitude Rossby waves (Feng et al. 2017; Nikumbh et al. 2023; Annamalai et al. 2023; Rana et al. 2019), and local moisture activity from the equatorial Indian ocean region (Rana et al. 2019; Abid et al. 2020). These regional variabilities are often modulated or triggered by climate modes such as ENSO, North Atlantic Oscillation (NAO), East Atlantic/West Russia (EA/WR) pattern and Indian Ocean Dipole (IOD) (Mehmood et al. 2022; Roy and Singh 2024).

Among these climate modes, ENSO has the most prominent footprint on HMA wintertime precipitation as revealed by studies mostly based on multilinear regression, Principal Component Analysis and linear correlations (Abid et al. 2020; Mehmood et al. 2022; Massoud et al. 2023). Present understanding suggests that the warm (cold) phase of ENSO strengthens (weakens) the subtropical jet stream in the winter season which drives stronger (weaker) storm tracks activity towards the HMA region (Yadav et al. 2009; Kamil et al. 2019; Roy and Singh 2024). Specifically, El Niño can warm up the tropospheric air temperature and create a north–south temperature gradient between the tropics and midlatitudes (Goswami and Xavier 2005). This leads to strengthening of the vertical zonal wind shear (∂u/∂z), equivalent to a thermal wind generation in a baroclinic instability scenario (Holton and Hakim 2013). This essentially means the strengthening of the subtropical westerly jet in the presence of El Niño and vice versa for La Niña. Secondly, the upper atmospheric divergence branch of ENSO triggers a Rossby wave train (Hoskins and Karoly 1981; Sardeshmukh and Hoskins 1988) that can reach over HMA (Rana et al. 2019; Annamalai et al. 2023). Usually, the upper-level divergence anomaly during ENSO can create a barotropic instability which can trigger meandering Rossby wave disturbances by conserving the absolute vorticity of the air column. These waves travel along the midlatitude jet stream across the Eurasian region reaching the HMA region at subtropical latitudes (Feng et al. 2017; Nikumbh et al. 2023). For instance, ENSO-associated Rossby Wave Source (RWS) (Sabatani and Gualdi 2025) developing near the gulf of America can trigger Rossby wavetrains directed towards north Atlantic-European (NAE) region and further to the east. In addition to the higher latitude response of ENSO, its influence on SST in the Indian ocean (Ashok et al. 2003; Cai et al. 2005; Jung and Kirtman 2016) and moisture transport towards HMA (Abid et al. 2020) have also been widely acknowledged. In this regard, given that El Niño (La Niña) triggers a low-level divergence (convergence) pattern over the maritime continent, this pattern could act as a means of transporting any available moisture from the equatorial Indian ocean towards north. Based on the above physical arguments, we hypothesize three plausible ENSO teleconnection pathways to HMA winter precipitation, namely: an extratropical wave pathway (EWP), a subtropical jet pathway (SJP), and a tropical moisture pathway (TMP) (Fig. 1a), and use a robust data-driven (nonparametric) causal discovery framework to test these hypotheses.

It is worth noting that subseasonal-to-seasonal (S2S) forecasting of HMA winter precipitation remains a challenge, even with state-of-art dynamical models such as NASA’s GEOS-S2S (e.g., Massoud et al. 2023). In this context, given that dynamical forecast models typically capture ENSO conditions well, a theory-guided causal teleconnections network of ENSO to HMA precipitation combined with domain-specific knowledge, is expected to reveal intermediate thermodynamic/dynamical processes responsible for HMA precipitation variability, offering the possibility to improve S2S predictive power in this hazard prone HMA region. Moreover, such an analysis could provide valuable diagnostic tools for comparing and improving physics-based models.

The rest of the paper is structured as follows. In Sect. 2 we present the PCMCI + methodology, followed by the three physically plausible hypothesized teleconnection pathways and the observables used to establish the precursors (Sect. 3). In Sect. 4, we apply the PCMCI + algorithm to test the causality of the proposed teleconnection pathways, and in Sect. 5 we compute the path coefficients of the individual causal links and quantify the causal effect strength of ENSO on HMA precipitation. Conclusions and a brief discussion are presented in Sect. 6.

## Methods

There are several causal discovery methods (Pearl 2009; Glymour et al. 2019; Zanga et al. 2022) widely used to infer causality. These include transfer entropy (Schreiber 2000), Granger causality (Granger 1969; Shojaie and Fox 2022), Graphical Models (Pearl 1988; Elwert 2013; Runge et al. 2015; Glymour et al. 2019), and Convergence Cross Mapping (Sugihara et al. 2012), among others; see also Hannart et al. (2016). The graphical PCMCI algorithm (Peter and Clark Momentary Conditional Independence) (Runge et al. 2015, 2019), has been widely used in climate studies and comparatively has been shown to be effective (Ebert-Uphoff and Deng 2012; Ombadi et al. 2020; Docquier et al. 2024) in dealing with highly dependent multivariate time series (Di Capua et al. 2020; Carvalho-Oliveira et al. 2024). Here, we apply PCMCI + (Runge 2020), a modified version of the PCMCI algorithm (Runge et al. 2015, Runge 2018) which accounts for both lagged and contemporaneous (lag-0) causal links in the multivariate climate data and also improves upon false negatives (Runge 2020, Karmouche et al. 2023).

In order to select inputs for the PCMCI + algorithm, oceanic and atmospheric observables relevant to the key mechanisms in each hypothesized pathway are first identified. Then, potential precursors are defined as regions wherein the observables exhibit significant linear correlation with HMA precipitation. The PCMCI + consists of two principal phases: a skeleton discovery phase and an orientation phase. In the skeleton discovery phase, the causal parents (lagged and contemporaneous) of all precursors are identified with the Peter and Clark (PC) causal algorithm (Spirtes et al. 2000) and the Momentary Conditional Independence (MCI) testing (Runge et al. 2015). In the PC step, the lagged parents of each precursor are identified from the initial set of lagged precursors through iterative conditional independence testing. Then, the Momentary Conditional Independence (MCI) test is performed on each precursor, conditioned on their contemporaneous (lag-0) parents and on the lagged parents, resulting in the final sets of causal parents of each precursor. Essentially, the skeleton discovery phase of PCMCI + removes spurious correlations for highly interdependent multivariate time series data and brings out the true causal links establishing the causal network structure. Both the PC and MCI steps can accommodate linear or nonlinear conditional independence testing (Runge et al 2019). Here we adapt a partial correlation-based PCMCI + test under the assumption of linear dependency among the multivariate climate time series.

In the orientation phase, the lagged links orient forward in time. For the contemporaneous links, the algorithm follows partial correlation-based orientation rules (see more on Runge 2020) that determine whether a link is orientated or undirected (Hastie et al. 2009). In the undirected case, one could take the decision to assign orientation based on robust physical knowledge of the system and comparing autocorrelation values of adjacent nodes. The result is a causal network graph (later in Fig. 4) that summarizes the causal dependencies, link strengths and time lags. There are two parameters needed to be assigned when the PCMCI + causal discovery test is performed and these are, the time lag the algorithm should consider for each observable and the statistical significance level (α) at which the PC and MCI tests are performed. In our case, we assign time lag = 2 (months) and significance level α = 5% for the causal discovery test. It is emphasized that it is crucial to combine the causal network discovery methodology with physical domain knowledge to pose and interpret physically meaningful teleconnection pathways.

## Setting-up testable hypotheses for causal pathways

In this section, we present carefully selected observables that we hypothesize are associated with the different physical mechanisms involved in each of the pathways and provide statistical evidence that the precipitation in HMA exhibits dependency on those observables. Over the 70-year period, none of the observables showed significant trends at 5% level, except SST in the eastern equatorial Pacific (of 0.07 K/decade), and over equatorial Indian ocean (0.11 K/decade). Accordingly, these trends are removed, and causal discovery analysis is performed on the resulting detrended and standardized (mean removed and divided by the standard deviation) time series of SST and the rest of the observables.

### Common elements across the proposed pathways

The three hypothesized teleconnection pathways share two elements: firstly, and obviously, the target (precipitation in HMA), and also, their common origin, the ENSO region. For the target variable, HMA precipitation (PR), we focus on the region of HMA with elevation higher than 1 km (Figs. 1b and S1) between 29°–43.25° N and 62°–78° E (~ 2.4 million km^2^) as this region receives the highest precipitation amount in winter (~ 310 mm on average). While reliable monthly precipitation records over HMA are available since 1901, we choose the 1951–2020 period for better accuracy and matching the availability of global monthly ERA5 reanalysis data (0.5° × 0.5°) for the atmospheric variables. We analyze monthly precipitation averages over the HMA region from the UK CRU vTS4.08 (0.5° × 0.5°) observational gridded global precipitation dataset over the period 1951–2020 (Harris et al. 2020). For SST, we use monthly sea surface temperature (SST) at 1° × 1° resolution from the UK MetOffice HAD-SST v3.2 (Rayner et al. 2003).

**Fig. 2:**
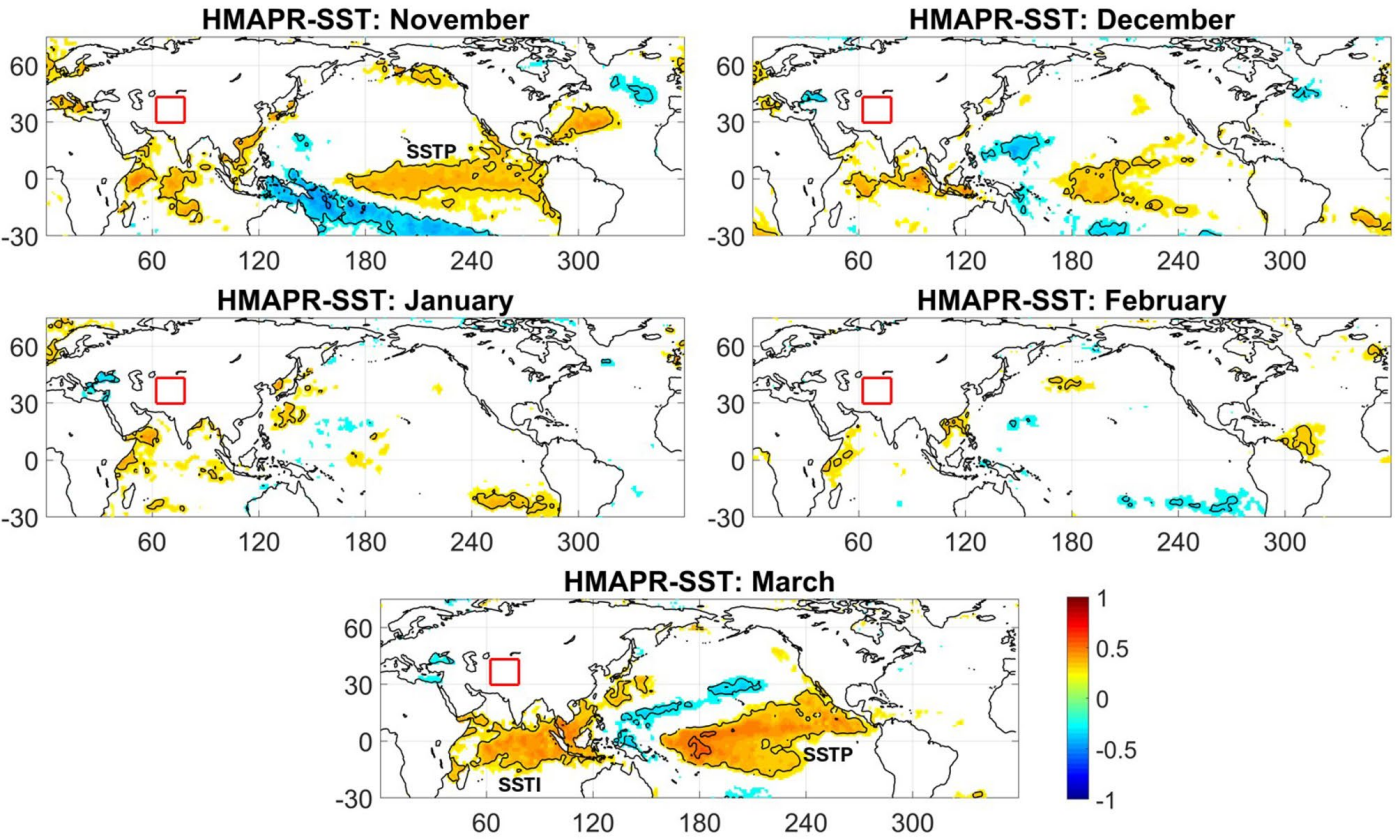
ENSO precursor regions to HMA winter precipitation: Linear correlation maps of HMA precipitation to global SST at lag-0 during November-March, revealing significant ENSO footprints in November and March (precursor regions labeled as SSTP for the months of November and March) and its diminishing presence during the mid-winter season. For the month of March, a significant precursor emerges also in the Indian ocean SST (labeled as SSTI). Correlation patterns are shown at 5% statistical significance level (See Fig. S2 for lagged correlation maps of HMA precipitation to global SST)

In order to confirm the ENSO influence, we first assess whether the Equatorial Pacific SSTs indeed act as a precursor to HMA winter precipitation (HMA PR). The linear correlation between HMA monthly winter precipitation and global SST at lag-0 (contemporaneous), evaluated at 5% significance level (Fig. 2), shows a positive correlation over the eastern equatorial Pacific in November, which becomes stronger in March (Fig. 2). The correlation remains strong at lag-1 and lag-2, i.e. September/October SSTs for the November PR (Fig. S2a–c), and January/February for the March PR (Fig. S2m-o). The positive correlation regions in the equatorial Pacific at lag-0,1,2 are the ENSO precursors to HMA precipitation in our analysis and are individually referred to hereafter as the ENSO region or SSTP (Figs. 2 and S2a–c and m–o). The correlation of HMA PR in November to the area-weighted SSTP at lag-1 is a moderate 0.37, and in March 0.41 (Fig. S3). For March PR, we further identify a strong SST precursor region over the equatorial Indian ocean. We denote this region as SSTI (Fig. 2) and include it for our causal discovery analysis for March. With respect to the other winter months, in December, the spatial extent and intensity of the correlation between equatorial Pacific SST and HMA PR diminishes (Figs. 2 and S2d-f) and becomes negligible for January–February precipitation (Figs. 2 and S2g-l). Based on this reduced and/or negligible correlation (also consistent with the absence of a significant relationship between HMA PR and the CPC NOAA Niño3.4 index—see Fig. S4), we focus our analysis on November and March HMA PR to test our ENSO-driven causal teleconnection hypotheses. In Sect. 4.3 (and section S1; Figs. S10–S12 in the supplementary material) we provide insight on the breakdown of the ENSO–HMA PR causal pathways during the December–February period.

As we observe significant precipitation to SST correlations up to lag-2, the PCMCI + analysis is carried out at an assigned maximum lag of 2 months and all causal links and strengths are tested at 5% significance level.

### Extratropical wave pathway (EWP)

**Fig. 3:**
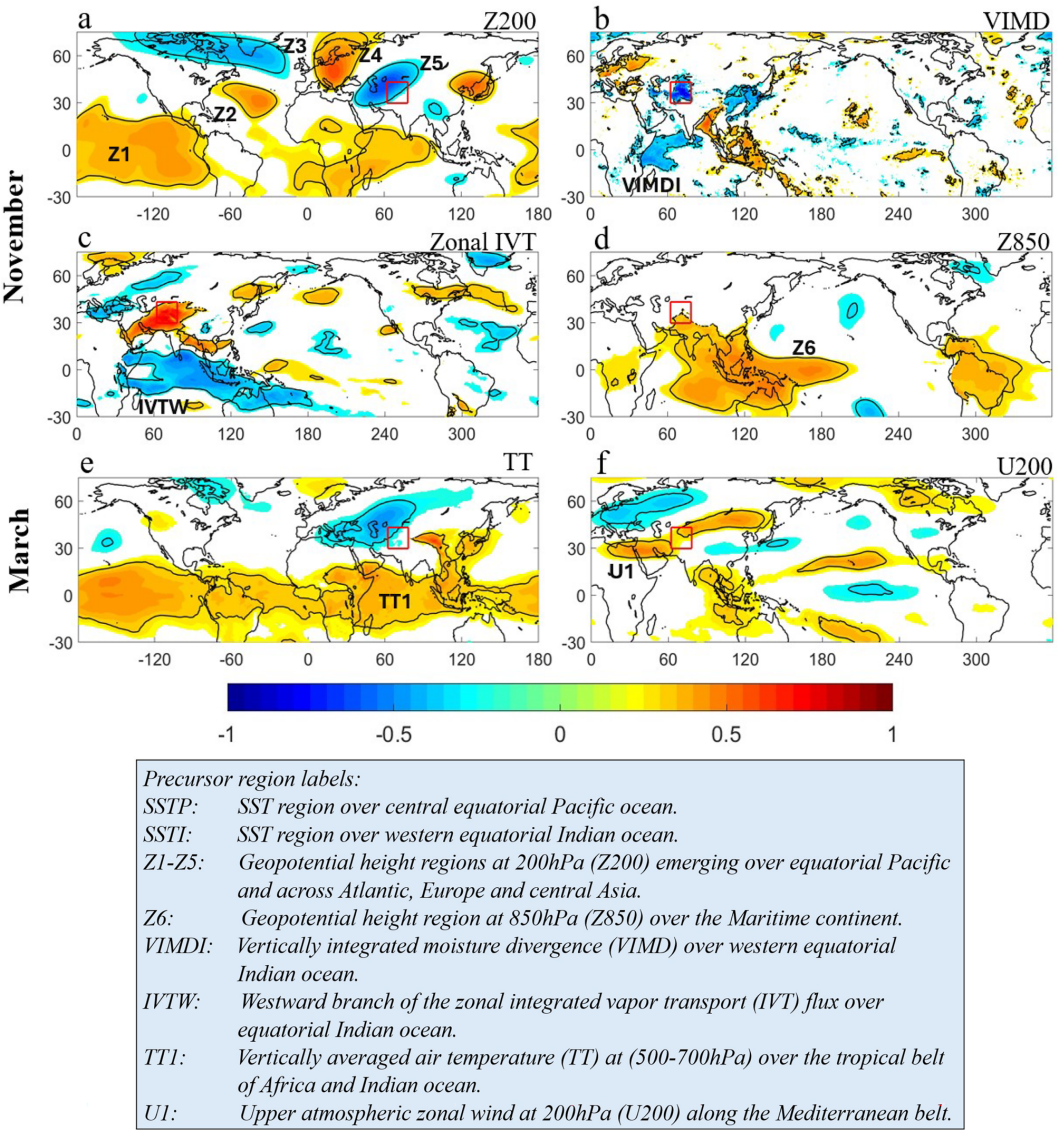
Precursor regions of atmospheric observables as potential causal parents of the ENSO teleconnection pathways to HMA precipitation: Precursors (based on correlation with HMA precipitation) for November as **a** Rossby wave response (Z200: Z1, Z2, Z3, Z4, Z5), **b** moisture convergence (VIMD: VIMDI), **c** zonal moisture transport (IVT:IVTW), **d** lower level circulation (Z850: Z6) and similarly key precursors for March as **e** tropospheric temperature (TT, 500–700 hPa: TT1), **f** subtropical westerly jet strength (U200: U1). The correlation patterns are shown at 5% significance level. A detailed description of all precursors of precipitation is given in the box above

The hypothesized Extratropical Wave Pathway is driven by a Rossby wave response (see Fig. 1a) caused by the upper-atmospheric branch of ENSO. We chose as observable the geopotential height at 200 hPa (Z200) from ERA-5 global reanalysis (Hersbach et al. 2020), which generally provides an appropriate measure of any atmospheric wave response. Figure 3a shows the Z200 precursor regions, identified by linear correlation analysis of Z200 with HMA November precipitation, resembling a Rossby wave teleconnection pattern along with other precursor regions over the Indian ocean and east Asia. Here, the Z200 precursor region over equatorial Pacific (positive correlation region marked as Z1 in Fig. 3a) is the upper-level ENSO response and the Z200 precursor region northwest of HMA (negative correlation region marked as Z5 in Fig. 3a) is the local Rossby wave influence over HMA, implying a plausible causality. Thus, we choose as relevant precursors for November HMA precipitation all the Z200 regions (labeled as Z1, Z2, Z3, Z4, and Z5) with significant correlation which appear to be part of the wave train pathway (Fig. 3a). A similar correlation analysis was performed using March PR as the target, but no relevant Z200 precursors signifying a Rossby wave signature were found, which discards the possibility of a causal EWP control in March PR over the HMA region.

### Subtropical jet pathway (SJP)

This hypothesized Subtropical Jet Pathway involves the effect of ENSO on tropospheric temperature (TT), which modulates the subtropical westerly jet and storm tracks activity towards the HMA region (see also discussion in the Introduction). Essentially, ENSO induced warming (cooling) of the tropical atmosphere over Africa and western Indian ocean would imply a positive (negative) temperature gradient from the tropics to midlatitude across the Mediterranean belt. This would trigger a vertical zonal wind shear leading to strengthening (weakening) of upper-level subtropical westerly jet strength, i.e. higher (lower) storm activity towards HMA region. Hence, we chose as observables for the SJP pathway the air temperature averaged at 500–700 hPa (TT) to capture the presence of a meridional temperature gradient, and the upper-level zonal wind (U200) to capture the subtropical westerly jet strength which drives the cyclonic storms mostly from the Mediterranean region towards HMA.

In March, we observe strong positive correlation of HMA precipitation with SST (Figs. 2 and S2m-o) over both Indian ocean (SSTI) and equatorial Pacific region (SSTP). The overwhelming ENSO response is evident (Fig. 3e) in terms of lower-level tropical warming (labeled as TT1) along with a negative correlation TT region northwest of HMA in the midlatitude (Fig. 3e). In addition, distinct positive correlation regions of U200 are observed along the subtropical jet axis (25° N–35° N) (Fig. 3f) west of HMA (labeled as U1). Hence, our hypothesis of a subtropical jet pathway (SJP) is plausible with the identified precursor signatures of the respective observables. The identified precursor regions for the PCMCI + test are then as follows: the Pacific SST as SSTP, Indian ocean SST as SSTI, tropospheric temperature (TT) over tropical Africa and western tropical Indian ocean as TT1 and zonal wind band along the subtropical jet axis as U1 (Fig. 3f). While the tropical TT regions are strongly correlated with ENSO, the negative TT region over central Asia (Fig. 3e) is not caused by ENSO (no significant correlation with SSTP at 5% level) and hence we choose not to include this as one of the precursors. As the SST represents the ENSO response in our SJP hypothesis, the TT precursor region over the equatorial Pacific (Fig. 3e) becomes another near surface temperature-based precursor and is considered redundant for our analysis. A similar precursor detection analysis for November under SJP hypothesis reveals no significant signals (Fig. S6) for the essential precursors such as tropospheric temperature and cyclonic storms and hence the SJP hypothesis is not applicable for November PR.

### Tropical moisture pathway (TMP)

This hypothesized Tropical Moisture Pathway (TMP) involves local Indian ocean moisture convergence, and Indo-Pacific lower-level divergence as means of transport, as discussed in the Introduction. We use vertically integrated moisture divergence (VIMD) as observable for Indian ocean moisture availability (negative VIMD), and geopotential height at 850 hPa (Z850) as observable for low level circulation patterns in the presence of ENSO. In November, we observe (Fig. 3b) strong negative correlation of HMA precipitation with VIMD (i.e. moisture convergence) over the western equatorial Indian ocean and also over the HMA region. While negative VIMD correlation over HMA is a local effect, the strong VIMD precursor region over the Indian ocean (VIMDI) is a sufficient indication to test the TMP hypothesis in November. Secondly, we observe a large positive correlation region of Z850 (Z6 in Fig. 3d) over the maritime continent extending up to the western equatorial Indian ocean. The sign and extent of the Z850 precursor is consistent with the low level divergence pattern in the west Pacific in the presence of ENSO enabling moisture transport along the Arabian coast. In addition, we observe strong correlation of HMA precipitation with zonal integrated vapor transport (IVT) (Fig. 3c), westward in the equatorial Indian ocean and eastward towards the HMA region. While VIMD and Z850 represent moisture availability and transport, the emergence of the westward IVT as an intermediate precursor between moisture and dynamical components further strengthens our TMP hypothesis to be tested for causality.

Thus, the identified precursor regions for the PCMCI + testing of the TMP hypothesis are (Figs. 2, 3 and S4): SST over the equatorial Pacific (SSTP), the lower-level divergence (Z850) region (Z6), westward moisture transport over the equatorial Indian ocean (IVTW), and moisture convergence over the western Indian ocean (VIMDI). We reiterate that all these precursors are considered at lag up to 2 (months) for the PCMCI + analysis.

A similar analysis for March shows no moisture convergence over the Indian ocean and thus no moisture to be transported towards HMA. Hence, no physical basis exists to test the causality of the TMP hypothesis for the March HMA precipitation. We have also looked at the key precursors of the respective pathways for El Niño and La Niña years separately (Fig. S7) during November and March to substantiate ENSO driven teleconnection patterns. Both phases of ENSO show robust teleconnections, with La Niña having stronger teleconnection patterns, which can be explained by the well-known El Niño-La Niña asymmetry (Yu and Kim 2011; Chen et al. 2022).

## Establishing causal pathways of ENSO to HMA precipitation

Now that the physically relevant precursors associated with each of the three pathways are established, we proceed with the causal attribution using PCMCI + analysis. The primary goal of the PCMCI + algorithm for each pathway is to assess whether there is a definite causal link between the ENSO SST region (SSTP) to HMA precipitation (PR), and identify those essential precursors that emerge as part of the causal network (causal parents) to establish the causal teleconnection pathway.

### HMA precipitation in November

As revealed by the above analysis, during the month of November only two of the hypothesized pathways emerged as having statistically significant precursors to proceed with causality testing. These pathways are the Extratropical Wave Pathway (EWP) and the Tropical Moisture Pathway (TMP). Below we independently test these two pathways for causality, followed by assessing their combined effect (EWTMPs), to determine the emergent causal network arising from the ENSO-driven extratropical Rossby wave and tropical moisture transport mechanisms influencing HMA November precipitation.

#### The extratropical wave pathway (EWP)

**Fig. 4:**
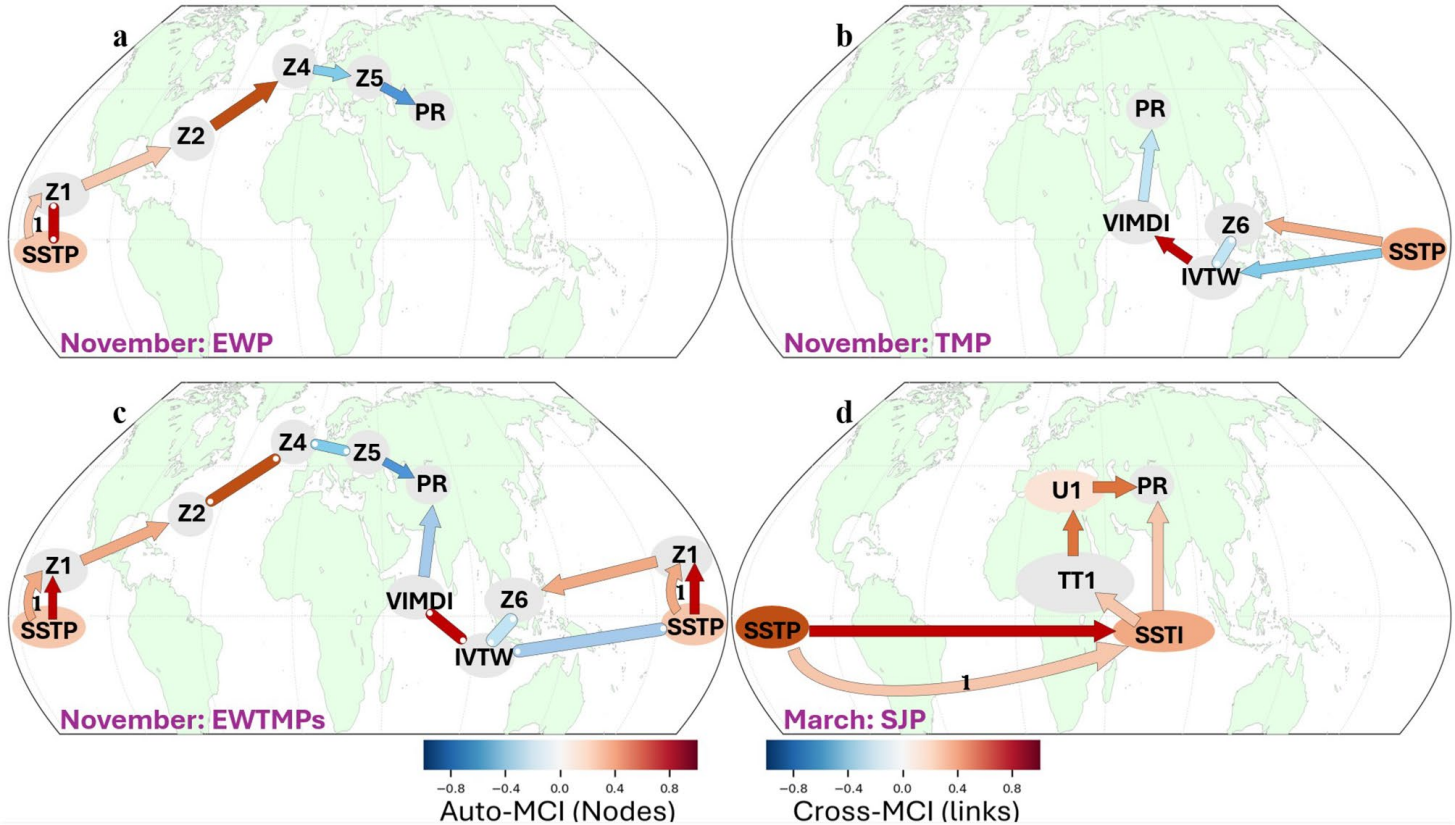
The causal network pathways of ENSO to HMA precipitation as emerged from the PCMCI + analysis shown on the global map: ENSO-driven causal networks of **a** extratropical wave pathway (EWP), **b** tropical moisture pathway (TMP), **c** emergent pathways under the combined influence of extratropical wave and tropical moisture (EWTMPs) for November precipitation, and **d** causal network of subtropical jet pathway (SJP) for March precipitation. The arrow color encodes link strength (bottom right colorbar; cross-MCI) and node color represents temporal autocorrelation (bottom left colorbar; auto-MCI). Straight arrows denote lag-0 links, whereas curved arrows indicate lagged links, with lags specified by labels. The results are based on PCMCI + test performed up to lag-2 and 5% significance level

As discussed in Sect. 3.2, the precursors of the EWP hypothesized pathway for November HMA precipitation are: SSTP (ENSO precursor over the equatorial Pacific) and the Z200 regions (Rossby wave precursors, Z1, Z2, Z3, Z4, and Z5) across the equatorial Pacific, Europe and central Asia (Figs. 2, 3a; see also the precursor time series in Fig. S5). The PCMCI + algorithm applied on these precursors indeed revealed a causally linked Rossby wave teleconnection pathway from the ENSO region to HMA region (Fig. 4a). Figure 4 shows the PCMCI + output (also as Fig. S8) that is overlaid on the global map to illustrate the locations of the causal parents. The immediate (adjacent) causal parent of PR emerged as Z5, the local upper atmospheric Z200 region northwest of HMA. Z5 was found to be causally linked to the ENSO precursor SSTP (Fig. 4a) via intermediate causal parents Z1, Z2 and Z4, which are evidently part of the wave train emanating from the ENSO region. The ENSO SST (SSTP) emerged as causally linked to its upper atmospheric response (Z1) via both a directed lag-1 link, and an undirected lag-0 link. Given the significant SSTP autocorrelation as shown by PCMCI + (Fig. 4a) which is near zero for the Z200 precursors (Z1, Z2, Z4, Z5), we can fairly argue that the lag-0 undirected link between SSTP and Z1 is directed as SSTP→Z1. Thus, Z5 emerges as the immediate causal parent of PR under the EWP pathway which is causally linked to ENSO via intermediate parents (Z1, Z2 and Z4) establishing the physically hypothesized extratropical wave pathway (EWP) (Figs. 1a and 4a). In physical terms, the warm (cold) phase of ENSO impacts HMA in November via an extratropical Rossby wave train resulting in increase (decrease) in precipitation.

The absence of Z3 as part of the causal network implies that while it is part of the wave train, the causal parent Z2 over the tropical Atlantic has mutual dependence on Z4, and therefore the correlation of Z3 with precipitation does not add new causal information about the physical mechanism. It is worth mentioning that we also tested PCMCI + with a limited set of precursors [SSTP, Z1 and Z5] and, it did not show a direct causal link between Z1 and Z5. This means that Z2 and Z4, as part of the wave train are essential to the causal network with significant mutual dependence on their adjacent parents and it further reinforces our EWP hypothesis. The EWP network also reveals that the auto-MCI scores of Z1, Z2, Z4 and Z5 nodes are near zero (Fig. 4a), implying that the wave train is triggered only in November and therefore is consistent with the 1–2 weeks timescale of a typical midlatitude Rossby wave train. The SSTP has relatively lower correlation with November PR (0.37) as compared to other parents like Z5 (0.60), and hence the absence of a direct causal link between SSTP and PR is also expected.

#### The tropical moisture pathway (TMP)

As detailed in Sect. 3.4, for the TMP hypothesis the following relevant precursor regions (Figs. 2, 3; see also Table S1 and Fig. S5) were identified from ocean-atmospheric correlation analysis with November PR in HMA: SSTP (ENSO precursor over equatorial Pacific), Z6 (lower-level circulation—Z850 over Maritime continent), VIMDI (moisture convergence over western equatorial Indian ocean) and IVTW (westward moisture transport over equatorial Indian ocean). The PCMCI + causal discovery algorithm was applied on PR, VIMDI, IVTW and SSTP and it revealed an ENSO-driven causal network (Fig. 4b) through the Indian ocean. The immediate causal parent of PR is VIMDI over the Indian ocean, where the negative causal link consistently indicates moisture convergence (or divergence) leading to higher (or lower) precipitation. The VIMDI is causally linked to equatorial Indian ocean transport (IVTW) which has two-way links to Indo-Pacific circulation (Z6) and ENSO (SSTP). Z6 is also causally linked to SSTP and establishes the overall network supporting the ENSO-driven TMP hypothesis. The VIMDI-IVTW link appears to be the strongest (mutual dependence) which is likely because both parents are a function of the moisture component, while the other parents are not. The causality of SSTP to PR via Z6, IVTW and VIMDI is consistent with the hypothesized moisture pathway. The two-way paths of SSTP to IVTW (Fig. 4b) indicate that ENSO can have both dynamic and thermodynamic effect over equatorial Indian ocean via local circulation (Z6) and zonal moisture transport (IVTW). Specifically, ENSO can transport the available Indian ocean moisture towards HMA region (Z6) and secondly, it can positively impact the moisture availability (VIMDI), both reinforcing the precipitation over HMA. While we hypothesize that ENSO only influences VIMDI via Indo-Pacific circulation, its direct impact on VIMDI actually bolsters our TMP hypothesis in November adding a new dimension to ENSO teleconnections. It is also consistent with existing studies of inter-basin influence of ENSO on Indian ocean SST (Abid et al. 2020; Wu et al. 2021) and thereby impacting low-level moisture development. In other words, ENSO-induced warming and lower-level circulation over the Indo-Pacific drives available moisture from the equatorial Indian ocean towards HMA.

#### Combined extratropical and tropical ENSO influence in November

It was revealed that the ENSO region exerts a two-way influence on HMA precipitation through an extratropical wave pathway (EWP) and a tropical moisture pathway (TMP). This raises the question: How independent are the two pathways and do the causal links to HMA precipitation identified for each pathway remain intact when both pathways are considered in tandem? To shed light on this question, we perform a new PCMCI + analysis considering as precursors the set of all the causal parents corresponding to each of the pathways (Di Capua et al. 2020). This union set of precursors consists of SSTP, Z1, Z2, Z4, Z5, Z6, IVTW and VIMDI (Table S1 and Fig. S5) and is used to assess the combined causal network in November. The PCMCI + analysis (Fig. 4c) reveals that both the ENSO-driven extratropical wave response (EWP) and tropical moisture transport (TMP) pathways retain their causal-link structures (Fig. 4a, b), when evaluated jointly, indicating that they provide complementary causal information and constitute mutually independent pathways influencing HMA PR. Minor modifications relative to the individual pathways are observed: (1) In the TMP branch, Z6 (Indo-Pacific divergence) is linked to SSTP (ENSO) via Z1 (local ENSO atmospheric response). This change does not alter the physical interpretation of the individual pathway and can be readily understood, by acknowledging that Z1 is not included as a precursor of TMP because it was not needed to test the hypothesized pathway; (2) Some causal links appear as undirected in the combined network, partly due to conditioning on a larger set of precursors which reduces statistical significance. However, given that these links exhibit consistent orientation in the individual causal networks and that such orientations are physically plausible, we retain that directionality in the combined network (Fig. 4c).

### HMA precipitation in March

#### The subtropical jet pathway (SJP)

Our linear correlation-based precursor analysis for March PR supported the hypothesized SJP to be further tested for causality. Particularly, the precursors identified (Figs. 2, 3 and Fig. S5) were the Pacific SST (SSTP), Indian ocean SST (SSTI), tropospheric temperature west of the tropical Indian ocean (TT1), and zonal wind along the subtropical jet axis (U1); see also Table S1. By applying the PCMCI + causal discovery algorithm, a robust causal network emerges (Fig. 4d) linking SSTP (ENSO) to HMA PR via two ways, one via the jet pathway (U1) and another via the Indian ocean SST (SSTI). U1 is linked to tropical warming (TT1) acting as a meridional temperature gradient which is then linked to regional Indian ocean SST (SSTI) at contemporaneous lag. The Indian Ocean SST is strongly linked to ENSO SST (SSTP) via lag-0 and lag-1 establishing the robust causal network supporting our SJP hypothesis. The ENSO again appears to have significant influence over the equatorial Indian ocean SST, which for the warm ENSO phase warms up the regional tropospheric temperature leading to strengthening of the subtropical jet and vice versa for the cold ENSO phase.

### Disruption of the ENSO-driven pathways to December–February HMA precipitation

As mentioned in Sect. 3.1, for the rest of the winter months (December–February), the correlation between HMA precipitation and SST over the ENSO region decreases in December (Fig. S2d–f) and is not significant in January and February (Fig. S2g–l), implying a negligible influence of ENSO. In this subsection, we further examine how the relevant pathways driving HMA PR are disrupted during the December–February period. Particularly, the analysis of the weakening of ENSO-HMA PR teleconnections during December-February (Figs. 2 and S10), following the dominant ENSO causal pathways in November (Fig. 4a-c), reveals that the extratropical wave (EWP) associated causal links break down across the tropical and North Atlantic regions, while the moisture transport (TMP) related causal links break down in the tropical Indian Ocean region (Figs. S11 and S12). As discussed in detail in section S1 (Supplementary Material), these findings are consistent with existing literature, which highlights ENSO dominance in November, followed by a more prominent influence of the North Atlantic Oscillation (NAO) and Tropical-West-East-Indian-Ocean (TWEIO) modes during December–February (Sabatani and Gualdi 2025), and ENSO influence on the phase reversal of NAO during December-February (Toniazzo and Scaife 2006; Jimnez-Esteve and Domeisen 2018; Geng et al. 2023). This may lead to different teleconnection outcomes towards our target HMA PR region even in the presence of ENSO. Hence, other climate modes such as, the NAO and TWEIO, may have a role in the possible disruption of ENSO teleconnections to HMA precipitation, as also supported by our analysis (Section S1). It is worth mentioning that we do see a remnant of the midlatitude wave-like pattern during December-February (Fig. S9), suggesting that HMA precipitation during those months might be subjected to the extratropical influence from NAO. However, the focus of our work is to investigate ENSO specific physically grounded causal pathways to HMA PR and hence exploring specific pathways of other climate modes falls beyond the scope of this study.

## Estimating the causal effect of ENSO on HMA winter precipitation

Having established causal networks, the PCMCI + algorithm allows us to quantify the strength of the causal effect of ENSO on the target variable (PR) based on the path coefficients that measure the strength of the individual causal links. The path coefficient of a causal link, say connecting nodes A→B, is the correlation coefficient (say β) determined by regressing the time series corresponding to the observable at node A on the time series corresponding to the observable at node B. Recalling that all the analysis is performed on the standardized time series (zero mean and 1 standard deviation SD), the magnitude of the path coefficient means that a 1 SD change in A would lead to a β SD change in B. Since PCMCI + reveals the causal parents of each variable (node) (Fig. 4) this allows us to estimate the path coefficients of all individual causal links, where the respective parent(s) of a node are the predictor(s) for the regression. Now, in a directed causal pathway composed of multiple intermediate parents, we multiply the path coefficients of the individual causal links (Pearl 2013; Runge et al. 2015) to estimate the total causal effect of the original parent (in our case ENSO) on the target variable (PR). If there exist multiple paths from the original parent to the target variable (PR), the sum of the product of the path coefficients of the individual paths would give the total causal effect on the target variable.

**Fig. 5:**
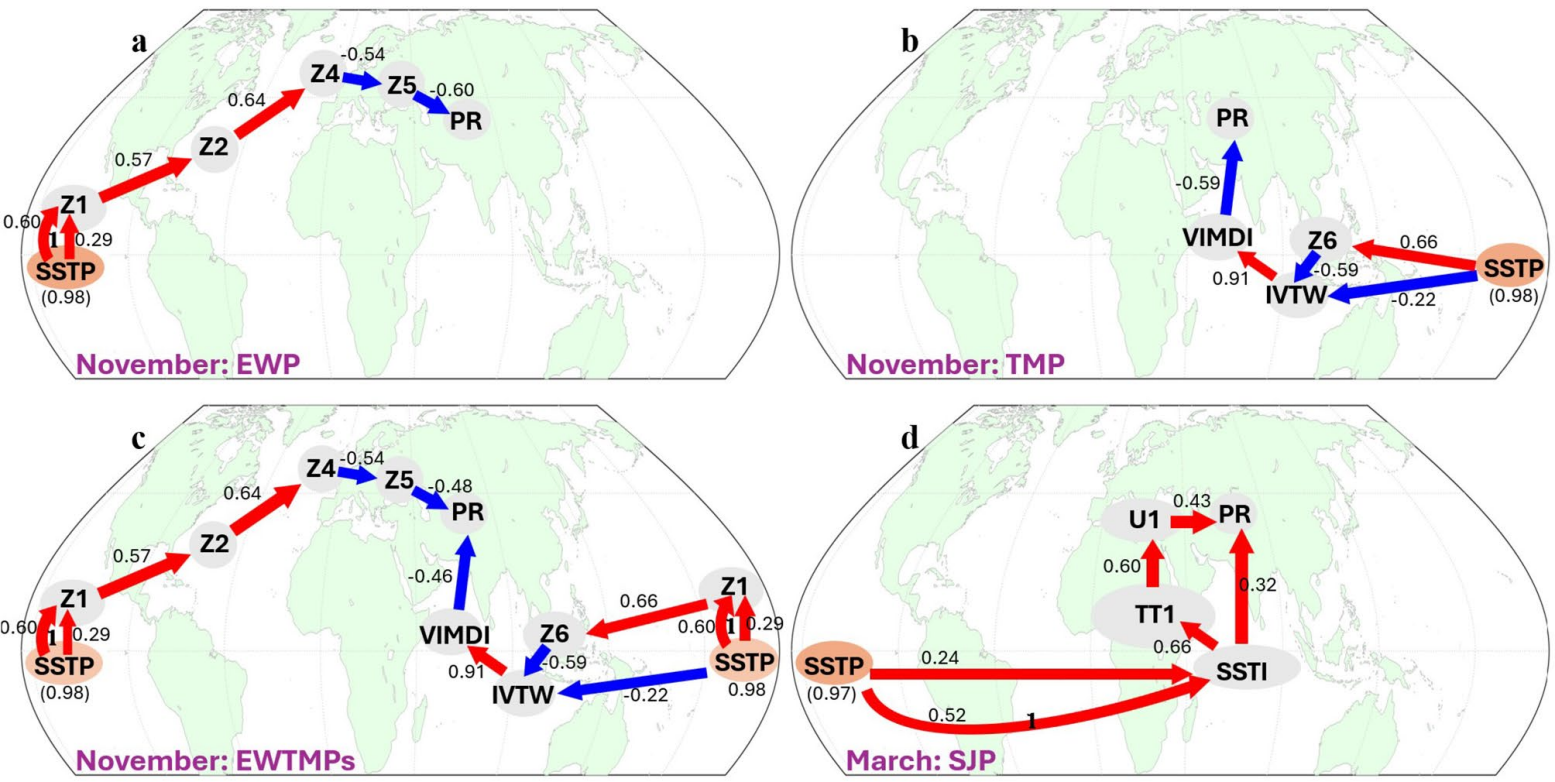
Path coefficients of PCMCI + based causal links for ENSO causal effect estimation on HMA PR: The path coefficients of individual causal links for **a** EWP **b** TMP **c** combined EWTMPs for November precipitation, and **d** SJP for March precipitation. The positive (negative) signs of the path coefficients are indicated by arrow colors red (blue) and, the path coefficients in parenthesis (below the SSTP node) are based on SSTP lag-1 auto-regression for the respective months

In November, we have shown that both the extratropical and tropical moisture pathways are active and thus the overall ENSO influence on HMA precipitation would be quantified by the causal effect coefficient computed from the combined EWTMPs pathways (Fig. 5c). We find that the mediated causal effect of ENSO via Z5 (extratropical way influence) in the combined pathway (SSTP→Z1→Z2→Z4→Z5→PR) is 0.08 (see detailed calculations in Table S2), and the mediated causal effect of ENSO through VIMDI (tropical moisture influence) is 0.23, which is the sum of the mediated causal effect through the SSTP→IVTW→VIMDI→PR and SSTP→Z1→Z6→IVTW→VIMDI→PR paths (Fig. 5c). Therefore, the total causal effect of ENSO on HMA November precipitation would be 0.08 + 0.23 = 0.31, i.e., a 1 SD change in ENSO in October would lead to a 0.31 SD change in precipitation in HMA in November. This clearly establishes the dominant role of ENSO as an external forcing in early HMA winter precipitation.

To provide extra insight into the influence of the two pathways acting independently, we also present the path coefficients computed for EWP and TMP separately. The causal network of EWP has only one path between ENSO (SSTP) at lag-1 and PR at lag-0 (Fig. 4a) with SSTP lag-1 autocorrelation coefficient of 0.98. Thus the total causal effect is the product of the path coefficients (Fig. 5a) of the individual causal links of the EWP, SSTP→Z1→Z2→Z4→Z5→PR, and this turns out to be 0.11. For TMP, overall causal effect of ENSO on PR is the sum of the causal effect of the two paths, SSTP→Z6→IVTW→VIMDI→PR and SSTP→IVTW→VIMDI→PR, which ends up being 0.32. This indicates a relatively stronger impact of ENSO through the moisture pathway as compared to Rossby waves which is also consistent with the estimated causal effects for the combined EWTMPs pathways shown above. It is noted that as both the EWP and TMP are simultaneously active in November, the Z5 and VIMDI turn out to be the adjacent causal parents of PR (Z5→PR←VIMDI). Hence, the path coefficients of the Z5 and VIMDI to PR links under the combined network (Fig. 5c) end up different from those obtained from the individual single pathway networks (Fig. 5a, b), in which there is only one adjacent causal parent of PR (Z5→PR for the EWP and VIMDI→PR for TMP).

In March on the other hand, ENSO influences HMA PR via the SJP pathway with two-way paths via the Indian ocean SST. The causal effect strength mediated via the subtropical jet (U1) SSTP→SSTI→TT1→U1→PR (Fig. 5d) is 0.13 and via the Indian ocean SST path SSTP→SSTI→PR is 0.24 (see Table S2 for computation). Hence the total causal effect of ENSO to March HMA precipitation is 0.24 + 0.13 = 0.37, i.e. 1 SD change in ENSO in February would lead to a 0.37 SD change in precipitation in March over HMA.

We recall that the linear correlation of SSTP at lag-1 to HMA PR is 0.37 in November and 0.41 in March (Sect. 3.1 and Fig. S3). Correlation however does not imply causation, and our causal discovery analysis established the actual cause-effect relationship of ENSO to HMA precipitation, restrained by physically reasoned mechanisms through appropriate intermediate processes. The causal effect strength computed through the causal networks (0.31 in November and 0.37 in March, which are slightly smaller than the corresponding correlation coefficients) shows consistency with what is expected from the path-tracing rule (Pearl 2013, Kretschmer et al. 2021), which states that, assuming the causal network identified by PCMCI + is causally sufficient, the sum of the products of the path coefficients along all causal pathways should approximately match the correlation coefficient between the parent (ENSO) and the target (HMA PR) variables. This agreement further substantiates the robustness of our physically grounded mechanisms of ENSO teleconnections to HMA PR.

As we know, climatologically, most of the winter precipitation in HMA is caused by storm track activity driven by the subtropical jet, especially when the influence of local midlatitude waves and tropical moisture availability are absent. So, any modulation of the jet strength by external forcing could lead to significant changes in HMA precipitation as we see here through the ENSO-driven SJP teleconnection. On the other hand, in November, tropical and subtropical pathways exert simultaneous influence on HMA precipitation leading to equivalent ENSO impact. Thus our findings establish, causally and quantitatively, the prominent role of ENSO on HMA precipitation variability in November and March, with significant implications for precipitation extremes and water resources management.

## Discussion and conclusions

In this study we have attempted to understand and causally establish the potential ENSO teleconnection pathways influencing HMA winter precipitation. Using a causal network framework, we have shown with 70 years of historical data, that ENSO has clear footprints on the early (November) and late (March) winter HMA precipitation, which constitutes about 45% of the total winter precipitation. We have established three ENSO-driven causal pathways, namely that ENSO drives an extratropical Rossby wave train (EWP) and tropical moisture transport (TMP) in November affecting HMA precipitation, while in March ENSO impacts HMA precipitation by modulating the subtropical westerly jet (SJP). The causal effect of ENSO on HMA PR is consistently comparable to their linear correlation but also physically reasoned, directed and quantified by data driven causal discovery tests. In November, the combined effect of EWP and TMP can lead to a 0.31 SD precipitation change in HMA region for 1 SD change in ENSO. In March, we see a strong ENSO correlation to HMA precipitation going back to lag-2 months and we establish a causal ENSO-driven subtropical jet pathway (SJP) with causal effect strength of a 0.37 SD precipitation change in HMA precipitation for 1 SD change in ENSO. For the rest of winter months (December-February), these pathways are disrupted, possibly due to the influence of other climate modes such as the North Atlantic Oscillation (NAO) and Tropical-West-East-Indian-Ocean (TWEIO) as discussed in Sect. 4.

We also note that our focus here has been on monthly precipitation, and therefore, some mechanisms operating at time scales shorter than a month might not necessarily lead to precursors at contemporaneous lags with precipitation. One example is the observations associated with Rossby waves, i.e., the Z200 regions, which capture the weekly propagation of the wave trains from west to east along the midlatitude jet streams towards HMA. However, it is noted that these Z200 regions were indeed identified as precursors at zero lag by linear correlation, and a subset of them were found to be casually linked to HMA precipitation by PCMCI + with oriented links, giving confidence in the robustness of the methodology and our results. In summary, the established ENSO-driven causal networks elucidate the historical state of ENSO-HMA precipitation teleconnections and can form the basis for improved subseasonal to seasonal (S2S) prediction, evaluation of climate models in terms of reproducing these teleconnections, and assessment of their possible change in response to changes in larger-scale climate modes, such as ENSO.

## Supplementary Information

Below is the link to the electronic supplementary material.

Supplementary file1

Supplementary file2

## Data Availability

All data including precipitation (UK-CRU, https://crudata.uea.ac.uk/cru/data/hrg/index.htm#current), Sea surface temperature (UK-Met Office, https://www.metoffice.gov.uk/hadobs/hadisst/data/download.html) and global atmospheric variables at single and pressure levels (ERA5, https://cds.climate.copernicus.eu/datasets) are taken from publicly available sources and also cited in references.

## Code availability

The causal discovery analysis and casual effect estimation are performed based on the Python package (Time Series Graph Based Measures of Information Transfer: TiGraMITE) by Jakob Runge which can be accessed through https://github.com/jakobrunge/tigramite. Other technical analysis and plotting are performed using MATLAB under UC Irvine subscription, and sample scripts are available at https://github.com/Pritamjborah/causal_ENSO_HMA.

## References

[CR1] Abid MA, Ashfaq M, Kucharski F, Evans KJ, Almazroui M (2020) Tropical Indian Ocean mediates ENSO influence over Central Southwest Asia during the wet season. Geophys Res Lett. 10.1029/2020GL089308

[CR2] Annamalai H, Neale RB, Hafner J (2023) ENSO-induced teleconnection: process-oriented diagnostics to assess Rossby wave sources and ambient flow properties in climate models. J Clim. 10.1175/JCLI-D-22-0346.1

[CR3] Ashok K, Guan G, Yamagata K (2003) Influence of the Indian Ocean dipole on the Australian winter rainfall. Geophys Res Lett. 10.1029/2003GL017926

[CR4] Cai W, Meyers G, Shi G (2005) Transmission of ENSO signal into Indian ocean. Geophys Res Lett 32:5. 10.1029/2004GL021736

[CR5] Cannon F, Carvalho LMV, Jones C, Norris J (2015) Winter westerly disturbance dynamics and precipitation in the western Himalaya and Karakoram: a wave-tracking approach. Theor Appl Climatol 125:27–44. 10.1007/s00704-015-1489-8

[CR6] Carvalho-Oliveira J, Di Capua G, Borchert LF, Donner RV, Baehr J (2024) Causal relationships and predictability of the summer East Atlantic teleconnection. Weather Clim Dyn 5:1561–1578. 10.5194/wcd-5-1561-2024

[CR7] Chen N, Fang X, Yu JY (2022) A multiscale model for El Niño complexity. Npj Clim Atmos Sci 5:16. 10.1038/s41612-022-00241-x

[CR8] Di Capua G, Runge J, Donner RV, van den Hurk B, Turner AG, Vellore R, Krishnan R, Coumou D (2020) Dominant patterns of interaction between the tropics and mid-latitudes in boreal summer: causal relationships and the role of timescales. Weather Clim Dyn 1:519–539. 10.5194/wcd-1-519-2020

[CR9] Docquier D, Di Capua G, Donner RV, Pires CAL, Simon A, Vannitsem SA (2024) Comparison of two causal methods in the context of climate analyses. Nonlinear Process Geophys 31:115–136. 10.5194/npg-31-115-2024

[CR10] Ebert-Uphoff I, Deng Y (2012) Causal discovery for climate research using graphical models. J Clim. 10.1175/JCLI-D-11-00387.1

[CR11] Elwert F (2013) Graphical causal models. In: Morgan SL (ed) Handbook of causal analysis for social research. Springer, Dordrecht, Neth, pp 245–273

[CR12] Feng J, Chen W, Li Y (2017) Asymmetry of the winter extra-tropical teleconnections in the Northern Hemisphere associated with two types of ENSO. Clim Dyn 48:2135–2151. 10.1007/s00382-016-3196-2

[CR13] Geng X, Noh KM, Kim K et al (2023) Midwinter breakdown of ENSO climate impacts in East Asia. Npj Clim Atmos Sci 6:155. 10.1038/s41612-023-00474-4

[CR14] Glymour C, Zhang K, Spirtes P (2019) Review of causal discovery methods based on graphical models. Front Genet 10:524. 10.3389/fgene.2019.00524 31214249 10.3389/fgene.2019.00524 PMC6558187

[CR15] Goswami BN, Xavier PK (2005) ENSO control on the South Asian monsoon through the length of the rainy season. Geophys Res Lett. 10.1029/2005GL023216

[CR16] Goswami BN, Chakraborty D, Rajesh PV et al (2022) Predictability of South-Asian monsoon rainfall beyond the legacy of Tropical Ocean Global Atmosphere program (TOGA). NPJ Clim Atmos Sci 5:58. 10.1038/s41612-022-00281-3

[CR17] Granger CWJ (1969) Investigating causal relations by econometric models and cross-spectral methods. Econometrica 37(3):424–438. 10.2307/1912791

[CR18] Hannart A, Pearl J, Otto FEL, Ghil M (2016) Causal counterfactual theory for the attribution of weather and climate-related events. Bull Am Meteor Soc. 10.1175/BAMS-D-14-00034.1

[CR19] Harris I, Osborn TJ, Jones P (2020) Version 4 of the CRU TS monthly high-resolution gridded multivariate climate dataset. Scientific Data 7:109. 10.1038/s41597-020-0453-3 32246091 10.1038/s41597-020-0453-3 PMC7125108

[CR20] Hastie T, Robert T, Jerome F (2009) The elements of statistical learning: data mining, inference, and prediction, Chapter 17, Second edition, Springer Press, https://link.springer.com/book/10.1007/978-0-387-84858-7

[CR21] Hersbach H et al (2020) The ERA5 global reanalysis. Q J R Meteorol Soc 146:730. 10.1002/qj.3803

[CR22] Holton J, Hakim GJ (2013) Introduction to dynamical meteorology, 5th Ed

[CR23] Hoskins BJ, Karoly DJ (1981) The steady linear response of a spherical atmosphere to thermal and orographic forcing. J Atmos Sci 38:6

[CR24] Hunt KMR, Baudouin JP, Turner AG, Dimri AP, Jeelani G, Chattopadhyay R, Cannon F, Arulalan T, Shekhar MS, Sabin TP, Palazzi E (2025) Western disturbances and climate variability: a review of recent developments. Weather Climate Dyn 6:43–112. 10.5194/wcd-6-43-2025

[CR25] Jimnez-Esteve B, Domeisen D (2018) The tropospheric pathway of the ENSO–North Atlantic teleconnection. J Clim 31:11. 10.1175/JCLI-D-17-0716.1

[CR26] Jung E, Kirtman BP (2016) ENSO modulation of tropical Indian Ocean subseasonal variability. Geophys Res Lett 43:24. 10.1002/2016GL071899

[CR27] Kamil S, Almazroui M, Kang IS (2019) Long-term ENSO relationship to precipitation and storm frequency over Western Himalaya–Karakoram–Hindukush region during the winter season. Clim Dyn 53:5265–5278. 10.1007/s00382-019-04859-1

[CR62] Karmouche S, Galytska E, Runge J, Meehl GA, Phillips AS, Weigel K, Eyring V (2023) Regime-oriented causal model evaluation of Atlantic–Pacific teleconnections in CMIP6. Earth Syst Dyn 14:309–344. 10.5194/esd-14-309-2023

[CR28] Kirschbaum D, Kapnick SB, Stanley T, Pascale S (2020) Changes in extreme precipitation and landslides over High Mountain Asia. Geophys Res Lett. 10.1029/2019GL085347

[CR29] Kretschmer M et al (2021) Quantifying causal pathways of teleconnections. Bull Am Meteorol Soc. 10.1175/BAMS-D-20-0117.1

[CR30] Lalande M, Ménégoz M, Krinner G, Naegeli K, Wunderle S (2021) Climate change in the High Mountain Asia in CMIP6. Earth Syst Dyn 12:1061–1098. 10.5194/esd-12-1061-2021

[CR31] Lyngwa RV, Hassan WU, Nayak MA, Azam MF (2023) Large fraction of winter precipitation variability in two major Himalayan basins explained by atmospheric rivers. J Clim. 10.1175/JCLI-D-22-0599.1

[CR32] Maina FZ, Kumar SV, Albergel C et al (2022) Warming, increase in precipitation, and irrigation enhance greening in High Mountain Asia. Commun Earth Environ 3:43. 10.1038/s43247-022-00374-0

[CR33] Massoud EC, Andrews L, Reichle R, Molod A, Park J, Ruehr S, Girotto M (2023) Seasonal forecasting skill for the High Mountain Asia region in the Goddard Earth Observing System. Earth Syst Dyn 14:147–171. 10.5194/esd-14-147-2023

[CR34] Mehmood S, Ashfaq M, Kapnick S et al (2022) Dominant controls of cold-season precipitation variability over the high mountains of Asia. Npj Clim Atmos Sci 5:65. 10.1038/s41612-022-00282-2

[CR35] Nash D, Carvalho LMV, Jones C (2022) Winter and spring atmospheric rivers in High Mountain Asia: climatology, dynamics, and variability. Clim Dyn 58:2309–2331. 10.1007/s00382-021-06008-z 35535316 10.1007/s00382-021-06008-z PMC9054897

[CR36] Nash D, Carvalho LMV, Rutz JJ (2024) Influence of the freezing level on atmospheric rivers in High Mountain Asia: WRF case studies of orographic precipitation extremes. Clim Dyn 62:589–607. 10.1007/s00382-023-06929-x 38274892 10.1007/s00382-023-06929-x PMC10806007

[CR37] Nikumbh AC, Thakur ABS, Chakraborty A, Bhat GS, Sukhatme J (2023) The role of the North Atlantic blocking high during large-scale heavy rainfall events over Central India. J Atmos Sci. 10.1175/JAS-D-22-0185.1

[CR38] Ombadi M, Nguyen P, Sorooshian S, Hsu K-L (2020) Evaluation of methods for causal discovery in hydrometeorological systems. Water Resour Res. 10.1029/2020WR027251

[CR39] Pearl J (1988) Probabilistic reasoning in intelligent systems: networks of plausible inference, 2nd ed. Morgan Kaufman Publishers

[CR40] Pearl J (2009) Causality, 2nd ed. Cambridge University Press

[CR41] Pearl J (2013) Linear models: a useful microscope for causal analysis. J Causal Inference 1(1):155–170. 10.1515/jci-2013-0003

[CR42] Rana S, McGregor J, Renwick J (2019) Dominant modes of winter precipitation variability over central southwest Asia and inter-decadal change in the ENSO teleconnection. Clim Dyn 53(9):5689–5707. 10.1007/s00382-019-04889-9

[CR43] Rayner NA, Parker DE, Horton EB, Folland CK, Alexander LV, Rowell DP, Kent EC, Kaplan A (2003) Global analyses of sea surface temperature, sea ice, and night marine air temperature since the late nineteenth century. J Geophys Res Atmos 108:D14. 10.1029/2002JD002670

[CR44] Roy S, Singh C (2024) The changing pattern of global teleconnection and the seasonal precipitation in the High Mountain Asia region. Clim Dyn 62:7665–7685. 10.1007/s00382-024-07300-4

[CR45] Runge J (2018) Causal network reconstruction from time series: from theoretical assumptions to practical estimation. Chaos 28:075310. 10.1063/1.5025050 30070533 10.1063/1.5025050

[CR46] Runge J, Petoukhov V, Donges J et al (2015) Identifying causal gateways and mediators in complex spatio-temporal systems. Nat Commun 6:8502. 10.1038/ncomms9502 26443010 10.1038/ncomms9502 PMC4633716

[CR47] Runge J, Nowack P, Kretschmer M, Flaxman S, Sejdinovic D (2019) Detecting and quantifying causal associations in large nonlinear time series datasets. Sci Adv. 10.1126/sciadv.aau4996 10.1126/sciadv.aau4996 PMC6881151 31807692

[CR48] Runge J (2020) Discovering contemporaneous and lagged causal relations in autocorrelated nonlinear time series datasets. In: Proceedings of the 36th conference on uncertainty in artificial intelligence (UAI), PMLR vol. 124, pp 1388–1397.

[CR49] Sabatani D, Gualdi S (2025) ENSO teleconnections with the NAE sector during December in CMIP5/CMIP6 models: impacts of the atmospheric mean state. Npj Clim Atmos Sci 8:226. 10.1038/s41612-025-01064-2

[CR50] Sardeshmukh PD, Hoskins BJ (1988) The generation of global rotational flow by steady idealized tropical divergence. J Atmos Sci 45:7

[CR51] Schreiber T (2000) Measuring information transfer. Phys Rev Lett 85:461. 10.1103/PhysRevLett.85.461 10991308 10.1103/PhysRevLett.85.461

[CR52] Shojaie A, Fox EB (2022) Granger causality: a review and recent advances. Annu Rev Stat Appl 9(1):289–319. 10.1146/annurev-statistics-040120-010930 37840549 10.1146/annurev-statistics-040120-010930 PMC10571505

[CR53] Song C, Huang B, Ke L, Ye Q (2016) Precipitation variability in High Mountain Asia from multiple datasets and implication for water balance analysis in large lake basins. Glob Planet Change 145:20–29. 10.1016/j.gloplacha.2016.08.005

[CR54] Spirtes P, Glymour C, Scheines R (2000) Causation, prediction and search. MIT Press

[CR55] Sugihara G, May R, Ye H, Hsieh C-H, Deyle E, Fogarty M, Munch S (2012) Detecting causality in complex ecosystems. Science. 10.1126/science.1227079 10.1126/science.1227079 22997134

[CR56] Toniazzo T, Scaife AA (2006) The influence of ENSO on winter North Atlantic climate. Geophys Res Lett 33:L24704. 10.1029/2006GL027881

[CR57] Wu X, Li G, Jiang W, Long S, Lu B (2021) Asymmetric relationship between ENSO and the Tropical Indian Ocean summer SST anomalies. J Clim 34(14):5955–5969. 10.1175/JCLI-D-20-0546.1

[CR58] Yadav RK, Kumar KR, Rajeevan M (2009) Increasing influence of ENSO and decreasing influence of AO/NAO in the recent decades over northwest India winter precipitation. J Geophys Res Atmos. 10.1029/2008JD011318

[CR59] Yoon Y, Kumar SV, Forman BA, Zaitchik BF, Kwon Y, Qian Y, Rupper S, Maggioni V, Houser P, Kirschbaum D, Richey A, Arendt A, Mocko D, Jacob J, Bhanja S, Mukherjee A (2019) Evaluating the uncertainty of terrestrial water budget components over High Mountain Asia. Front Earth Sci 7:120. 10.3389/feart.2019.00120 10.3389/feart.2019.00120 PMC7816802 33479598

[CR60] Yu J-y, Kim ST (2011) Reversed spatial asymmetries between El Niño and La Niña and their linkage to decadal ENSO modulation in CMIP3 models. J Clim. 10.1175/JCLI-D-11-00024.1

[CR61] Zanga A, Ozkirimli E, Stella F (2022) A survey on causal discovery: theory and practice. Int J Approx Reason 151:101–129 (**ISSN 0888-613X**)

