# Supplementary material for: The three causal pathways of ENSO teleconnections to High Mountain Asia winter precipitation
Source: Clim Dyn. 2026 Jun 1;64(6):267. doi: 10.1007/s00382-026-08198-w (PMC13226391; doi:10.1007/s00382-026-08198-w)
Supplement: Supplementary file 1 — Supplementary file1 [file 382_2026_8198_MOESM1_ESM.docx]

**Supplementary Information**

**The Three Causal Teleconnections Pathways of ENSO to High Mountain Asia Winter Precipitation**

Pritam Jyoti Borah^1^, Antonios Mamalakis^3,4^, Clement Guilloteau^1^_,_ Alejandro Tejedor^1,5,6^ & Efi Foufoula-Georgiou^1,2^

^1^Department of Civil and Environmental Engineering, University of California Irvine, Irvine, CA, USA

^2^Department of Earth System Science, University of California Irvine, Irvine, CA, USA

^3^Department of Environmental Sciences, University of Virginia, Charlottesville, VA, USA

^4^School of Data Science, University of Virginia, Charlottesville, VA, USA

^5^Institute for Biocomputation and Physics of Complex Systems (BIFI), University of Zaragoza, Zaragoza, Spain

^6^Department of Theoretical Physics, University of Zaragoza, Zaragoza, Spain

Corresponding author: Pritam Jyoti Borah ([borahp@uci.edu)](mailto:borahp@uci.edu))

**
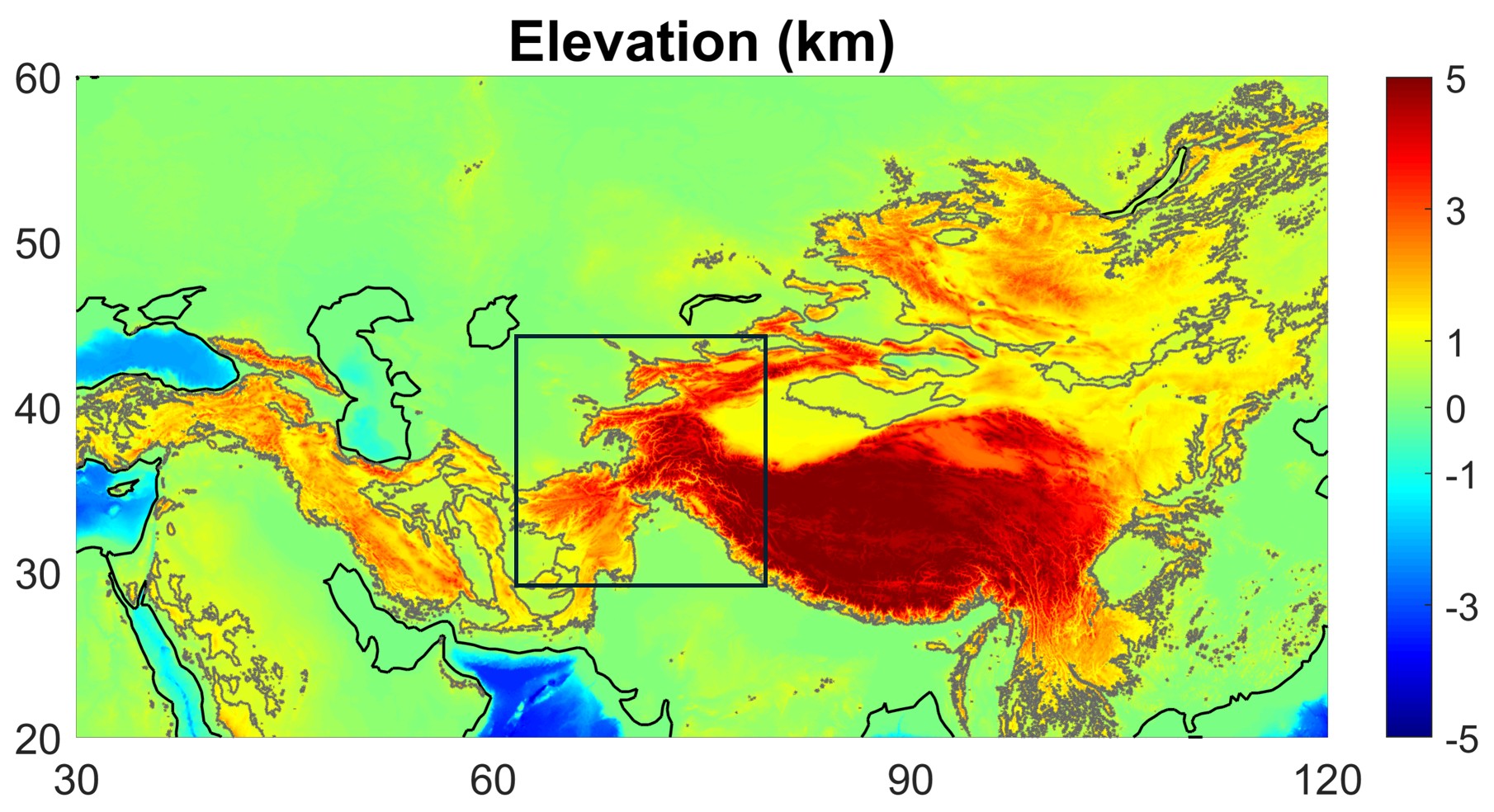
**

Figure S1: Terrain elevation map of Asia highlighting the high mountain region (grey contour at 1km). The high mountain Asia region in the black box is our region of interest in this study as this region receives the highest precipitation during the boreal winter (November-March) season, based on NOAA-NCEI ETOPO2 data (https://www.ncei.noaa.gov/products/etopo-global-relief-model).

**
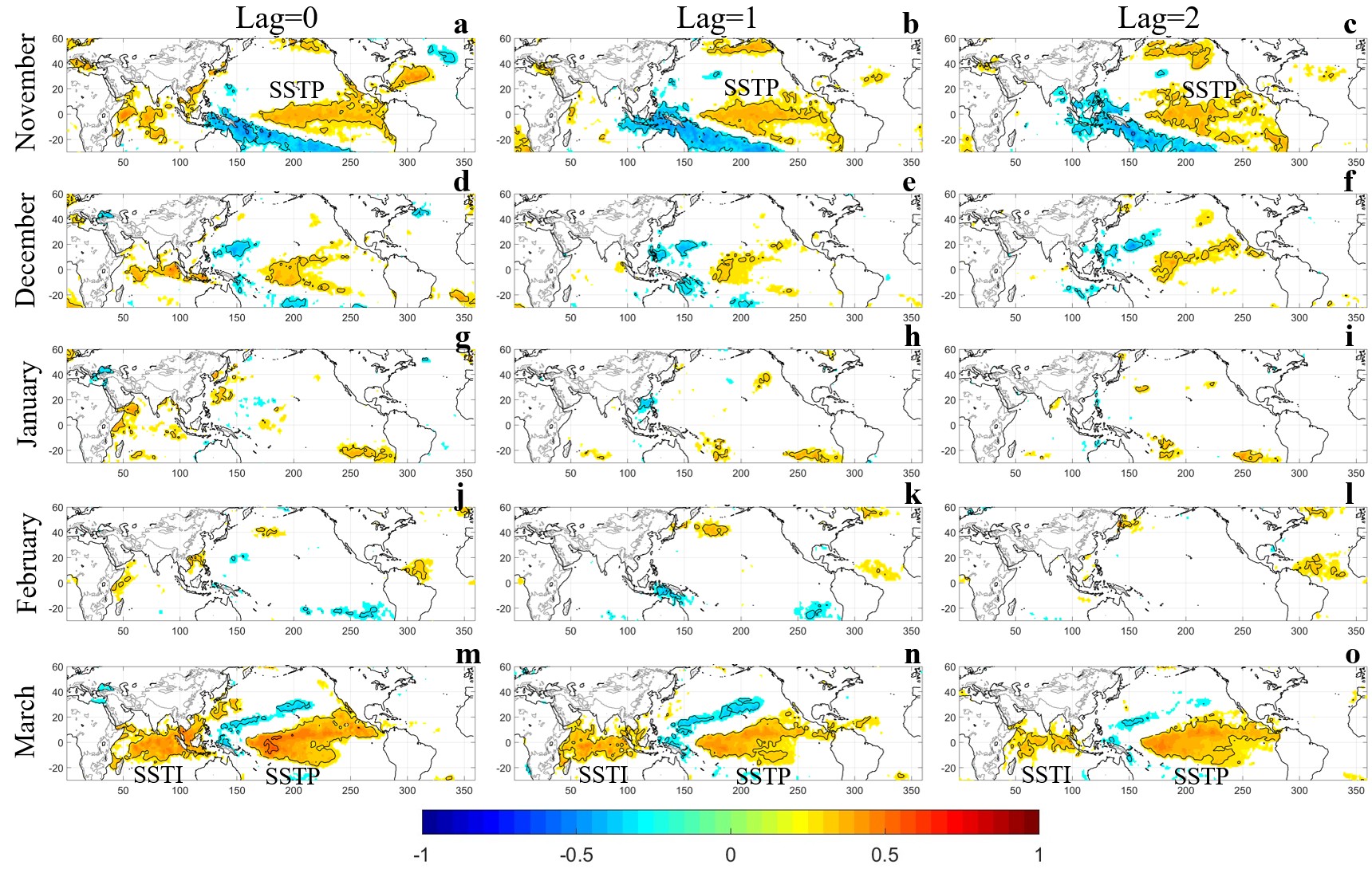
**

Figure S2: Correlation maps of area-weighted HMA precipitation time series to global SST during November to March at lag=0,1,2. The positive correlation regions over the equatorial Pacific for November and March are labelled as SSTP and over the Indian ocean for March as SSTI, and their area-weighted time series are used as the ENSO precursors for PCMCI+ testing. Correlation patterns are shown at 5% significance level.

**
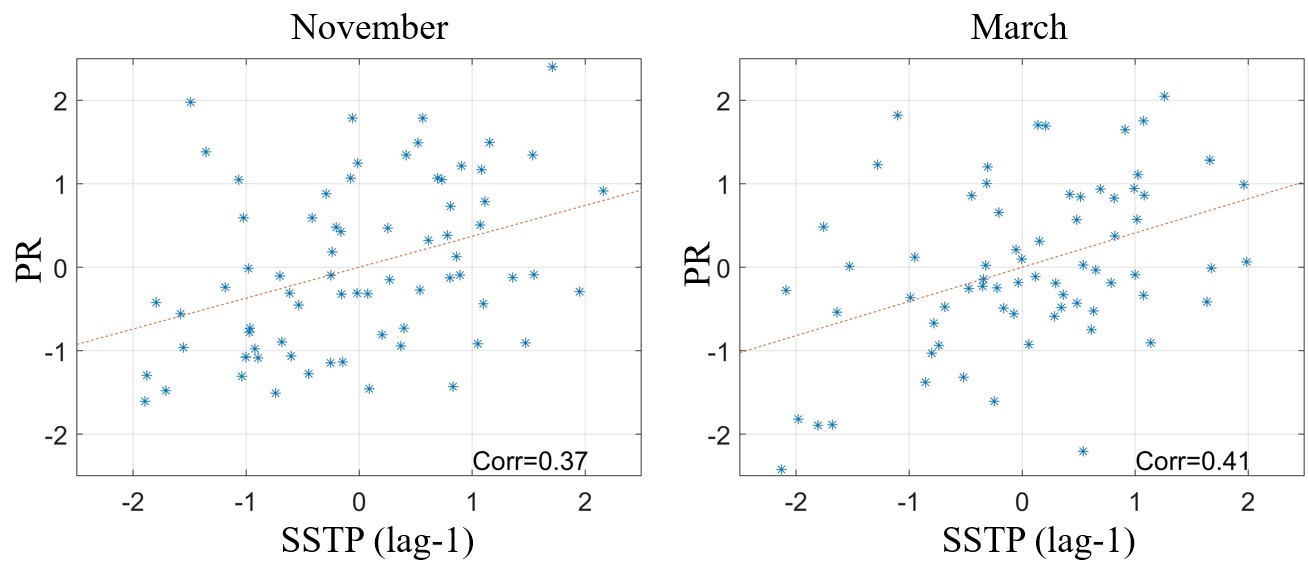
**

Figure S3: Scatter plots of HMA PR versus lag-1 SSTP in November and March showing direct relationship between ENSO and HMA PR, but without accounting for potential intermediate physical processes. The SSTP time series at lag-1 correspond to the area-weighted SST precursor region over the equatorial Pacific in November (Fig. S2b) and March (Fig. S2n).


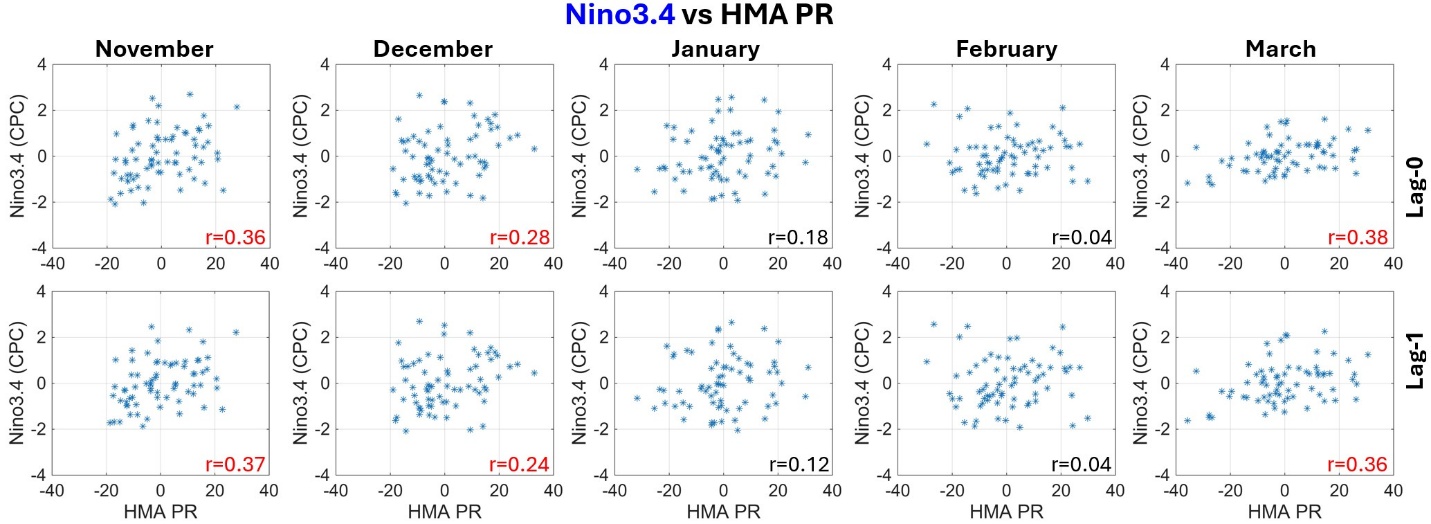


Fig. S4: Evolution of the ENSO-HMA PR relationship during the winter months: Scatter plots of NOAA CPC Nino3.4 index versus HMA PR during November-March at lag-0 (top row) and lag-1 (bottom row) for the 70 years of observations. The correlation coefficient (r) between HMA PR and Nino3.4 is shown in each panel, with values in red indicating statistical significance at 5% level and in black, indicating non-significant correlations. The correlation is significant during November, December and March, but not during January and February. These values are consistent with the correlation between HMA PR and the emerged SSTP precursor regions (Fig. 2, Fig. S2).


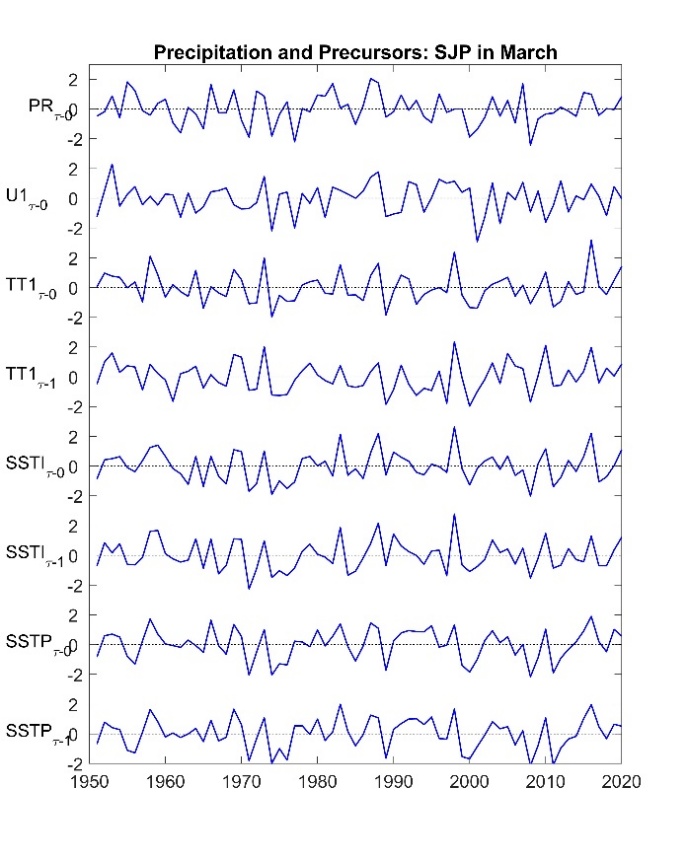

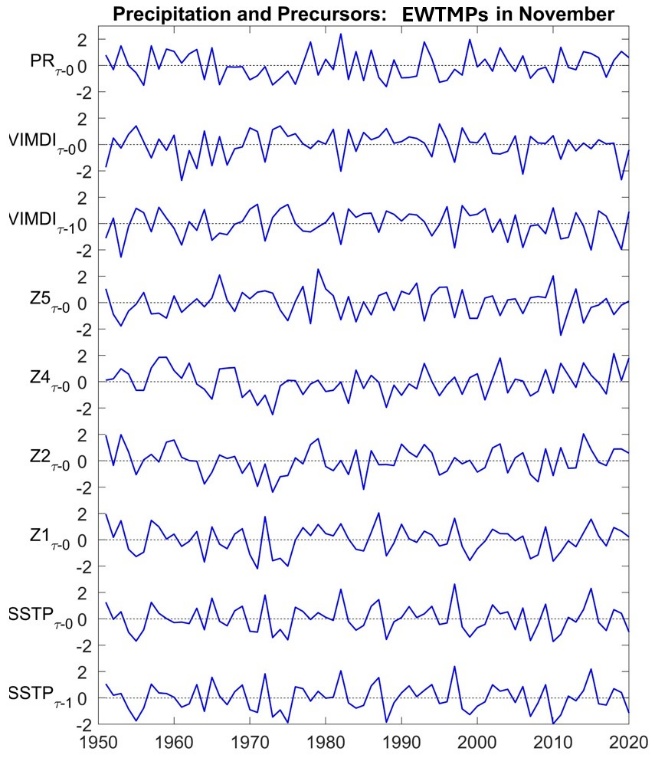

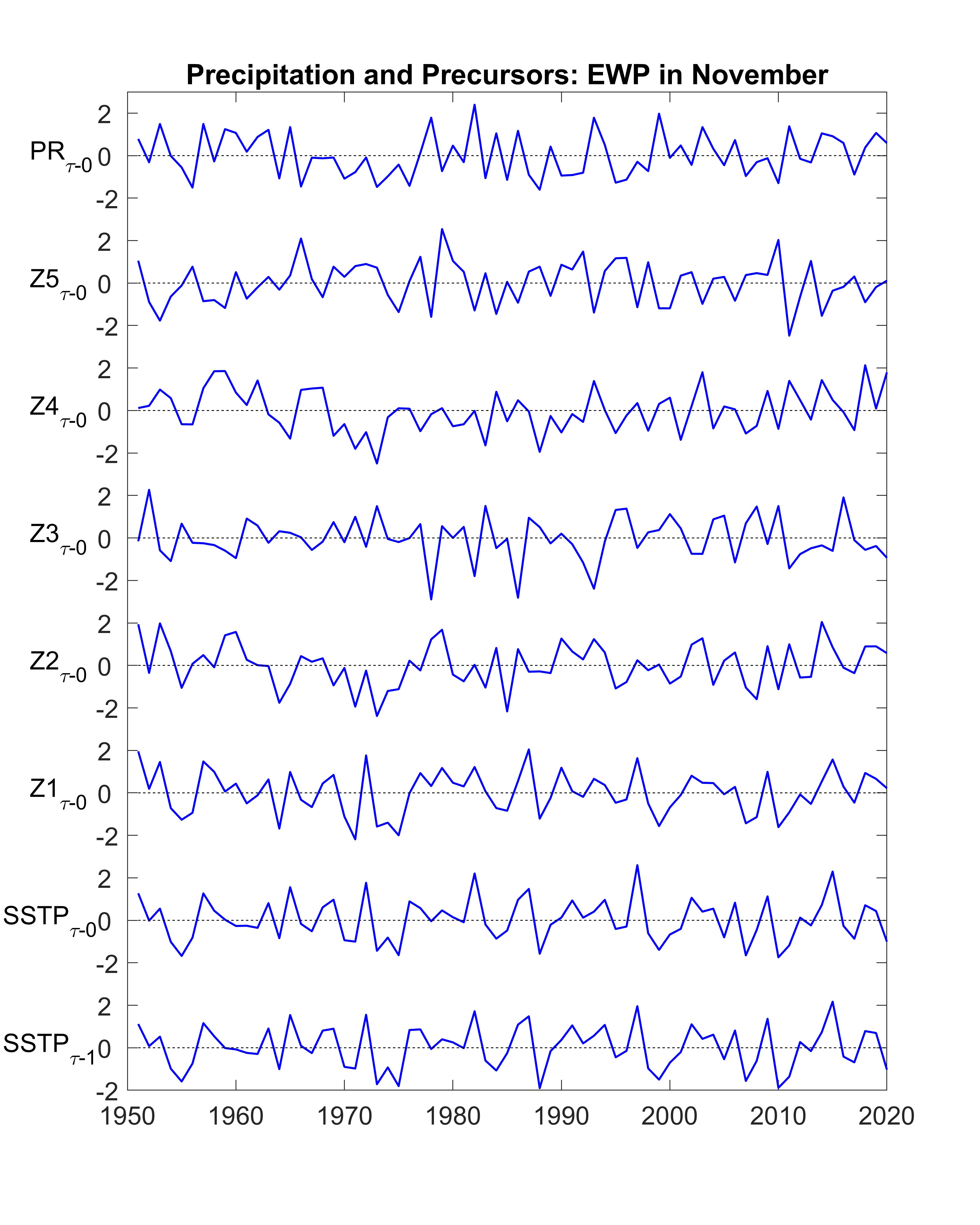

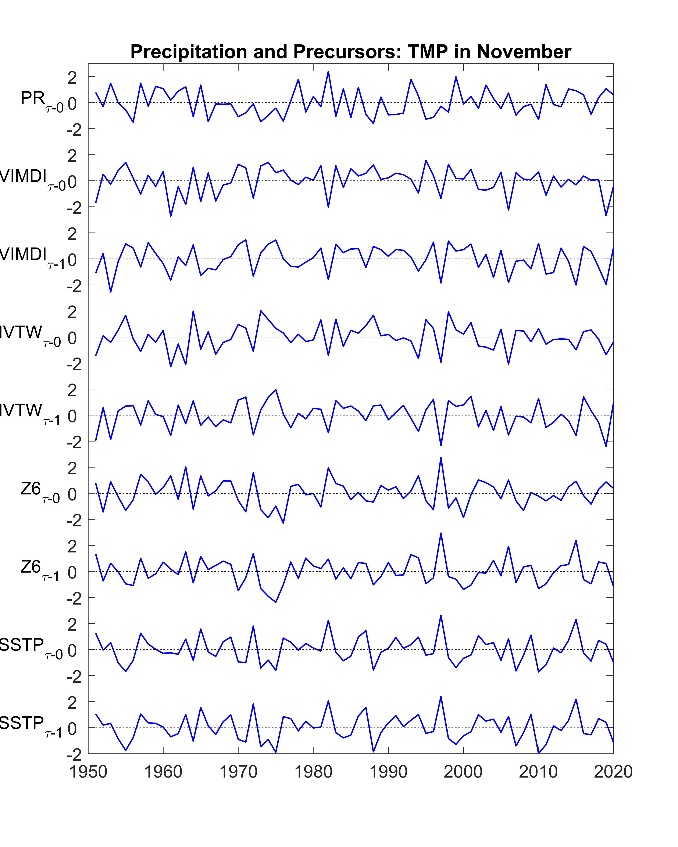


Fig. S5: Standardized anomaly time series of pixel area-weighted HMA precipitation and its precursors (at time lag τ) identified for EWP, TMP, and combined EWTMPs in November and SJP in March for the PCMCI+ causal discovery test.

| **November** | | | **March** |
| --- | --- | --- | --- |
| EWP | TMP | EWTMPs | SJP |
| PR Precursor regions identified based on correlation:  {SSTP, Z1, Z2, Z3, Z4, Z5}  Lag-0 correlation with HMA precipitation:  {Z5=-0.60, Z4=0.49, Z3=-0.53, Z2=0.53, Z1=0.41, SSTP=0.37} | PR Precursor regions identified based on correlation:  {SSTP, VIMDI, IVTW, Z6}  Lag-0 correlation with HMA precipitation:  {VIMDI=-0.59, IVTW=-0.56, Z6=0.53, SSTP= -0.37} | PR Precursor regions identified based on correlation:  {SSTP, Z1, Z2, Z4, Z5, Z6, IVTW, VIMDI}  Lag-0 correlation with HMA precipitation:  {Z5=-0.60, Z4=0.49, Z2=0.53, Z1=0.41, VIMDI=-0.59, IVTW=-0.56, Z6=0.53, SSTP=0.37} | PR Precursor regions identified based on correlation:  {SSTP, SSTI, TT1, U1}  Lag-0 correlation with HMA precipitation:  {U1=0.52, SSTP=0.48, SSTI=0.44, TT1=0.41} |
| *Precursor region labels:*  *SSTP: SST region over central equatorial Pacific Ocean.*  *SSTI: SST region over western equatorial Indian ocean.*  *Z1-Z5: Geopotential height regions at 200hPa (Z200) emerging over equatorial Pacific and across Atlantic,*  *Europe and central Asia.*  *Z6: Geopotential height region at 850hPa (Z850) over the Maritime continent.*  *VIMDI: Vertically integrated moisture divergence (VIMD) over western equatorial Indian ocean.*  *IVTW: Westward branch of the zonal integrated vapor transport (IVT) flux over equatorial Indian ocean.*  *TT1: Vertically averaged air temperature (TT) at (500-700hPa) over the tropical Africa and Indian ocean.*  *U1: Upper atmospheric zonal wind at 200hPa (U200) along the Mediterranean belt.* | | | |

Table S1: Summary of the precursors, parents and causal parents for ENSO-driven teleconnections to HMA precipitation: Extratropical wave pathway (EWP), Tropical moisture pathway (TMP) and combined EWTMPs in November, and Subtropical jet pathway (SJP) in March.


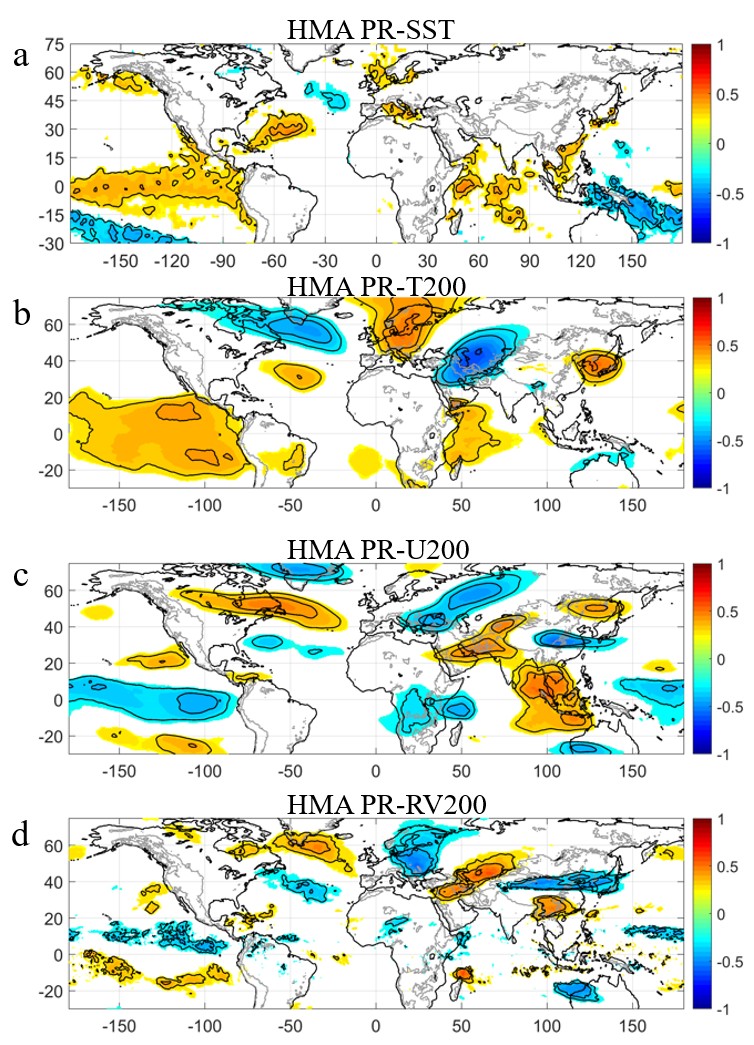


Figure S6: Correlation maps of SST and additional upper atmospheric observables with HMA Precipitation (PR) in November including (a) SST, (b) air temperature at 200hPa (T200), (c) zonal wind at 200hPa (U200) and (d) relative vorticity at 200hPa (RV200). The correlation patterns exhibit dominant signatures of the extratropical wave pathway (EWP) teleconnection and no sign of the subtropical jet pathway (SJP) teleconnection.

**
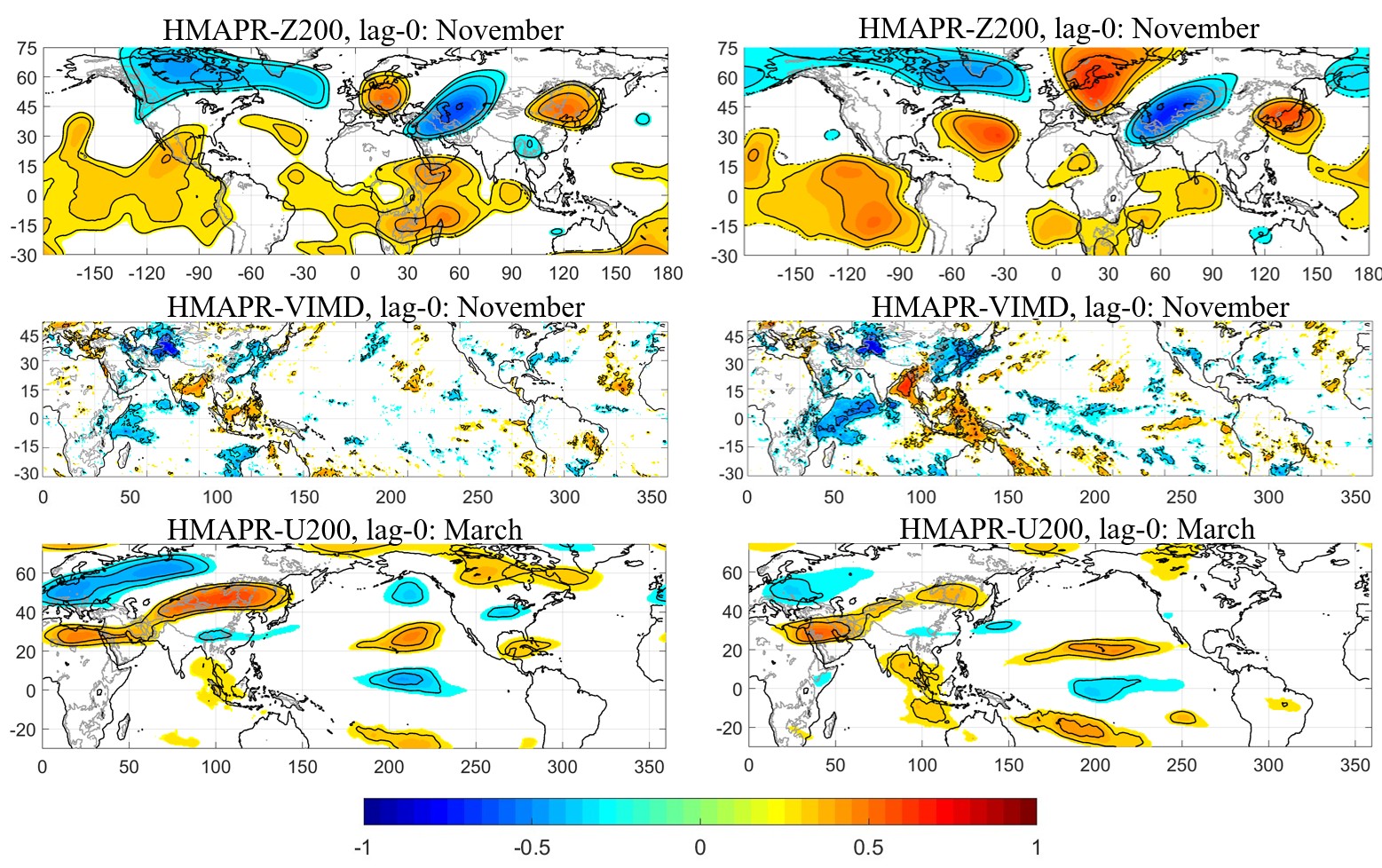
El Niño La Niña**

Figure S7: Key precursors of HMA precipitation for warm (El Niño) and cold (La Niña) phases of ENSO in November and March: Z200, VIMD precursors in November and U200 precursor in March. This shows that the teleconnection patterns are intact with both phases of ENSO but with an asymmetry in magnitude. El Niño and La Niña years are identified if the ONI (Oceanic Niño Index) is consecutively active during September-November for November precipitation and the same during January-March for March precipitation. ONI is 3 month running mean of ERSST.v5 SST anomalies in the Niño 3.4 region (5^o^N-5^o^S, 120^o^-170^o^W), where anomalies are based on a 30-year running mean. (https://origin.cpc.ncep.noaa.gov/products/analysis_monitoring/ensostuff/ONI_v5.php)


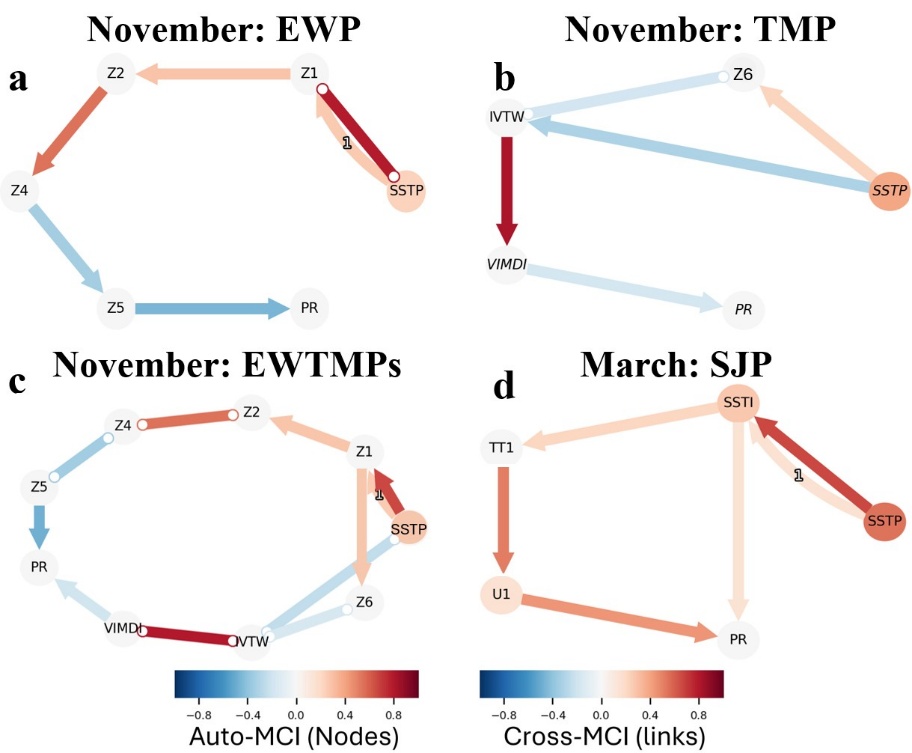


Fig. S8: PCMCI+ output of the causal networks of (a) the Extratropical Wave Pathway (EWP), (b) the Tropical Moisture Pathway (TMP), (c) the combined Extratropical Wave and Tropical Moisture Pathways (EWTMPs) in November and (d) the Subtropical Jet Pathway (SJP) in March. The PCMCI+ analysis was performed with τ_max_=2 (lag) at 5% significance level. The arrow colors indicate link strength (bottom right colorbar; cross-MCI) and node colors encode temporal autocorrelation (bottom left colorbar; auto-MCI). Straight arrows denote lag-0 links, whereas curved arrows indicate lagged links with lags specified by labels. Note that this figure displays the same information than Fig. 4, but without the background global map.


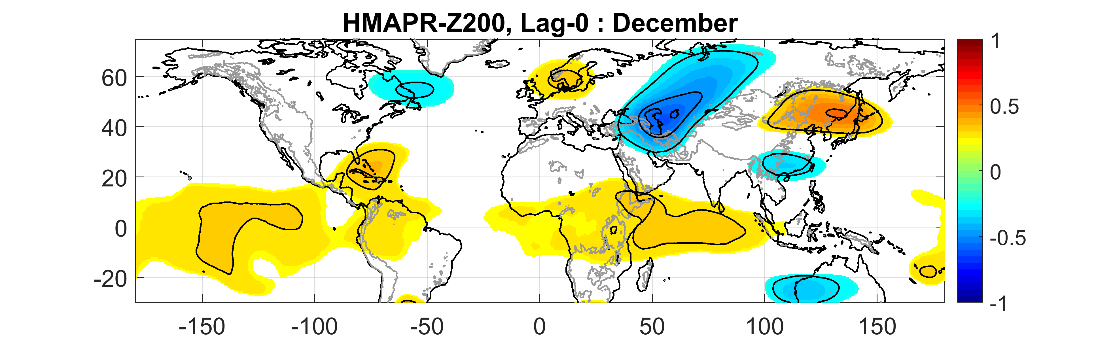


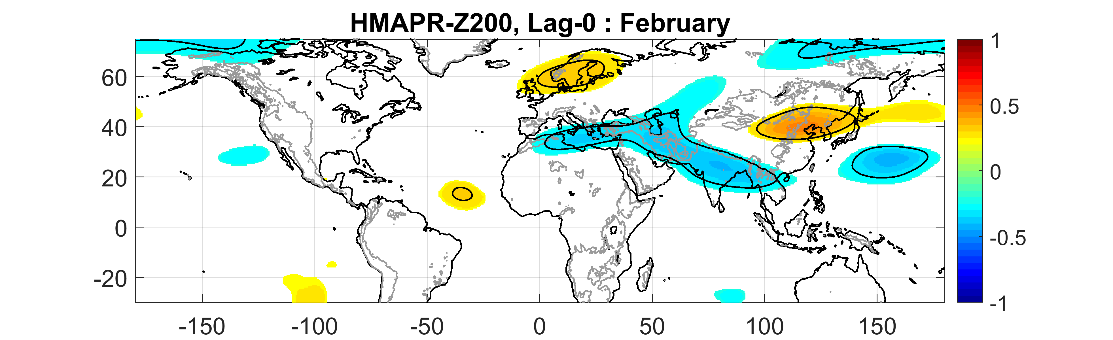

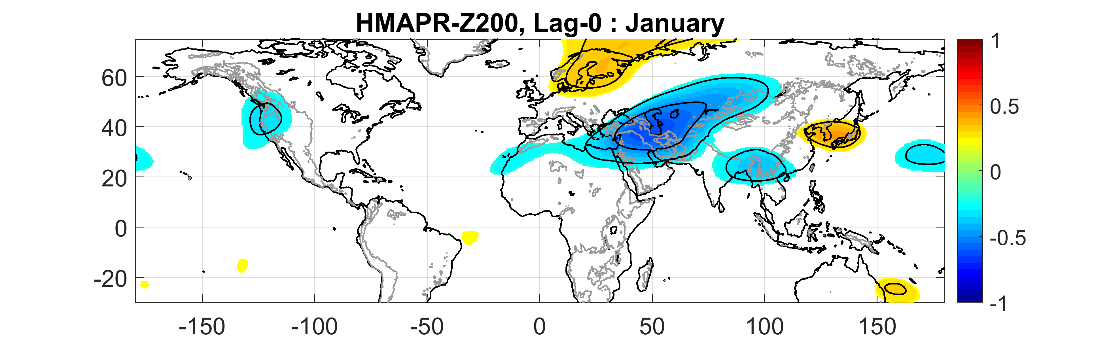


Figure S9: Correlation maps of HMA precipitation with global Z200 during December, January and February, indicating remnant of a Rossby wave like pattern probably originating from the North Atlantic region, which may be associated with NAO teleconnections.

| **November**, *Fig. 5a: Only Extratropical Wave Pathway (EWP) active* | |
| --- | --- |
| ENSO causal pathway via Z5  ENSO causal effect on PR | SSTP🡪Z1🡪Z2🡪Z4🡪Z5🡪PR  = [(0.29×0.98+0.60)×0.57×0.64×(-0.54)×(-0.60)] = 0.11, where 0.98 is the SSTP lag-1 autocorrelation coefficient. |
| **November**, *Fig. 5b: Only Tropical Moisture Pathway (TMP) active* | |
| ENSO causal pathways via VIMDI  ENSO causal effect on PR | SSTP🡪Z6🡪IVTW🡪VIMDI🡪PR & SSTP🡪IVTW🡪VIMDI🡪PR  = [0.98×(-0.59)×0.66×0.91×(-0.59)]+[0.98×(-0.22)×0.91×(-0.59)] = 0.32 |
| **November**, *Fig. 5c: Both Extratropical Wave Pathway + Tropical Moisture Pathway (EWTMPs ~~EWP+TMP~~) active* | |
| ENSO causal pathway via Z5  Mediated causal effect via Z5  ENSO causal pathways via VIMDI  Mediated causal effect via VIMDI  **Total ENSO causal effect on HMA PR in November** | (SSTP🡪Z1🡪Z2🡪Z4🡪Z5🡪PR)  = (0.29×0.98+0.60)×0.57×0.64×(-0.54)×(-0.48) = 0.08  SSTP🡪Z1🡪Z6🡪IVTW🡪VIMDI🡪PR & SSTP🡪IVTW🡪VIMDI🡪PR  = [(0.60+0.29×0.98)×0.66×(-0.59)×0.91×(-0.46)] + [(-0.22) ×0.98×0.91×(-0.46)] = 0.23  **= 0.08+0.23 = 0.31** |
| **March**, *Fig. 5d: Subtropical Jet Pathway (SJP) active* | |
| ENSO causal pathway via U1  Mediated causal effect via U1  ENSO causal pathway via SSTI  Mediated causal effect via SSTI  **Total ENSO causal effect on HMA PR in March** | (SSTP🡪SSTI🡪TT1🡪U1🡪PR)  (0.24×0.97+0.52)×0.66×0.60×0.43=0.13, where 0.97 is SSTP lag-1 autocorrelation coefficient.  SSTP🡪SSTI🡪PR  (0.24×0.97+0.52)×0.32 = 0.24  **= 0.13+0.24 = 0.37** |

*Table S2: Computation of the causal effect strength of ENSO on HMA PR in November and March based on the path coefficients as in Fig. 5.*

Section S1: ENSO-HMA PR Relationship during mid-winter


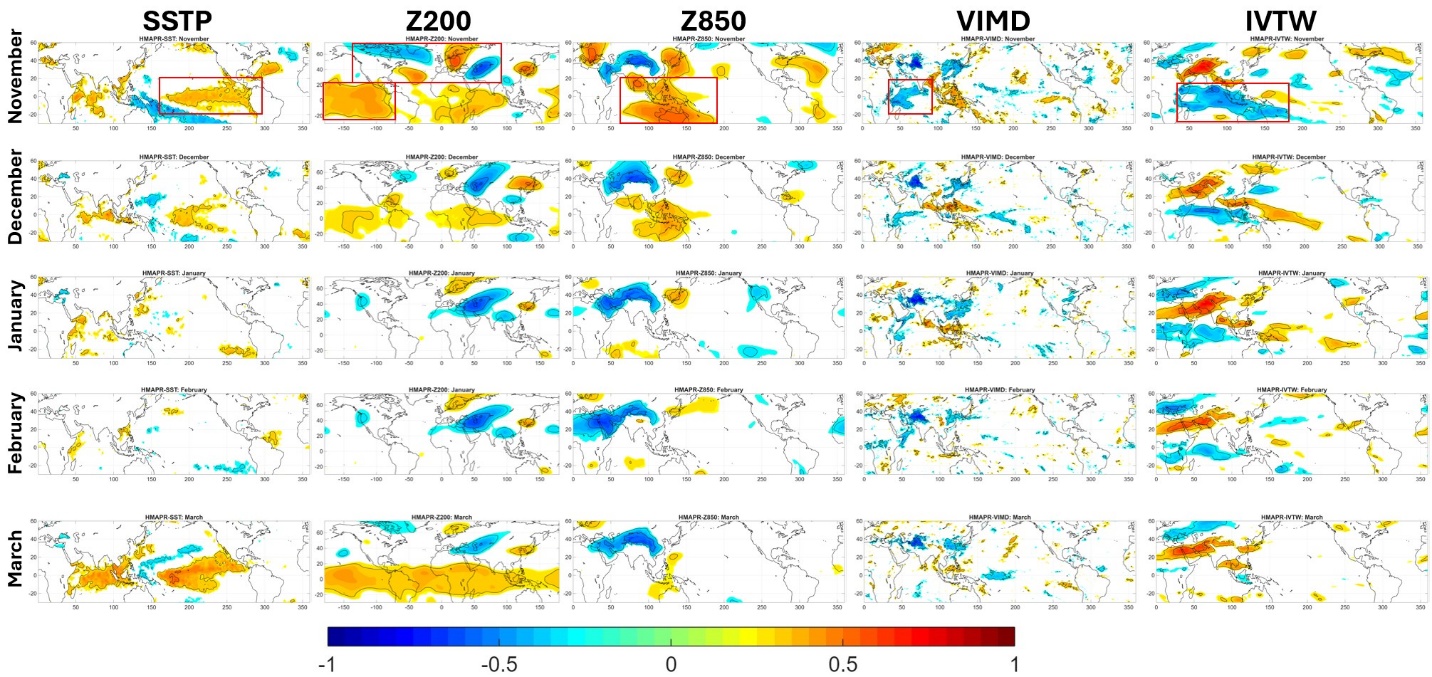
The evolution of the correlation between HMA Precipitation and global SST (Fig. 2, Fig. S2) shows that the SSTP precursor region over the equatorial Pacific decays during December-February (DJF). We further show (Fig. S10) that the significant precursor regions in November gradually shrink and vanish in subsequent months for the essential observables. Since the PCMCI+ algorithm relies on identifying significant dependencies to establish causal links, the absence of statistically significant precursors in mid-winter would, by default, result in a fragmented or empty causal network.

Fig. S10: Evolution of the EWP and TMP precursor regions of key observables with respect to HMA PR during November to March. The observables shown are SST and Z200 for EWP hypothesis, and SST, VIMD, Z850 and Zonal IVT (IVTW) for TMP hypothesis. The precursor regions (highlighted by the red boxes in November) weaken and vanish during the mid-winter (DJF) period just like the ENSO-HMA PR correlation.

Rather than simply acknowledging the lack of signal, we argue that significant insight can be gained by examining how and where the different pathways fail as the winter season progresses. For this we imposed the November precursor regions (location and shape) in all months and extracted for each month the precursor time series for each observable i.e., for SSTP, Z1, Z2, Z4, Z5, VIMDI, IVTW, Z6, and PR to proceed with PCMCI+ testing. This would allow us to understand the evolution of the emergent causal networks during DJF with respect to November.

From the PCMCI+ causal networks that emerged in the individual months (Fig. S11), we computed the path coefficients of the causal links as shown in Fig. S12. We observe distinct weakening and breakdown of the causal pathways for both EWP and TMP after November, which is evident from both the emergent causal networks (Fig. S11) and the evolution of the path coefficients (Fig. S12). For EWP (Fig. S12a), the Z1🡪Z2 (blue) and Z2🡪Z4 (green) causal links over the tropical Atlantic and north Atlantic gradually weaken and break down during the December to March period, leading to disruption of the ENSO driven causal pathway to HMA PR. For TMP (Fig. S12b), the causal links associated with moisture availability over the Indian ocean weaken after November and the pathway to the HMA PR breaks down. Specifically, the SSTP🡪IVTW (blue) and VIMDI🡪PR (red) links break down after November, whereas the IVTW🡪VIMDI (pink) link gradually weakens with the progression of winter. This is in agreement with the shrinking of the Z200 precursors across the Atlantic and the moisture convergence and transport (VIMD, IVTW) precursors over the tropical Indian ocean region (Fig. S10). Therefore, while ENSO may be active during DJF, the likely disruption of its teleconnections across the tropical Atlantic and the absence of moisture
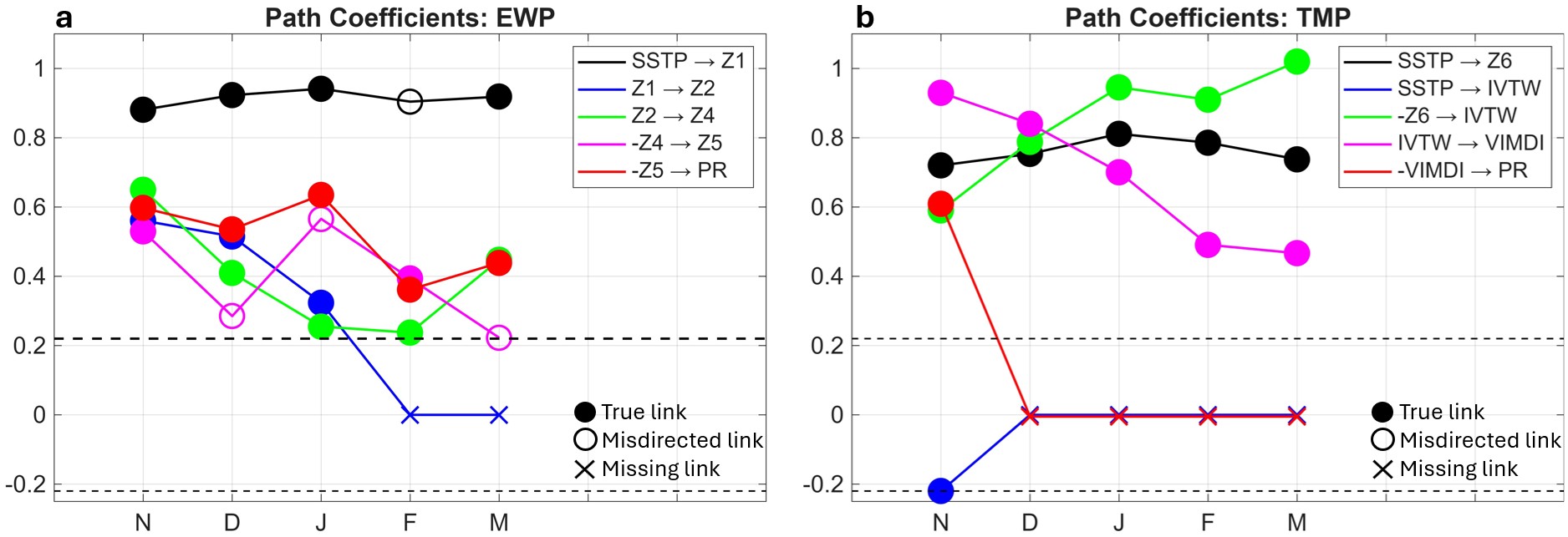

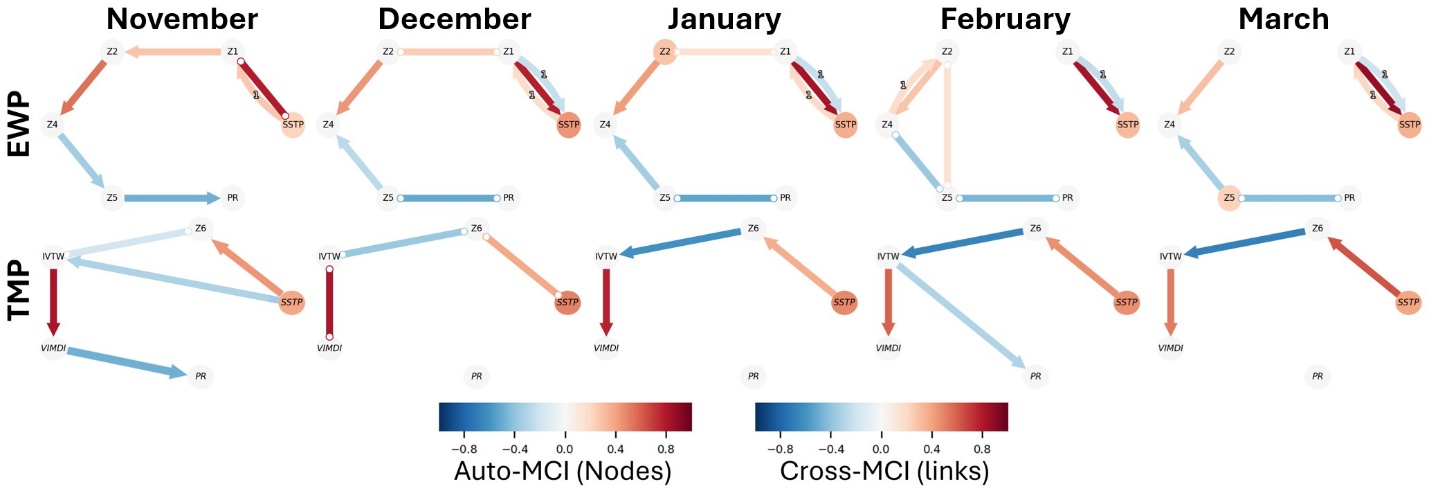
convergence (divergence) in the Indian ocean may have led to the causal link breakdown for both pathways.

Fig. S12: Evolution of the (a) EWP and (b) TMP path coefficients from November to March. ‘Solid circle’ denotes statistically significant path coefficients with p-value<0.05, ‘hollow circle’ denotes statistically significant path coefficient but with the wrong direction, and ‘cross’ denotes not statistically significant path coefficient (p-value>0.05); The broken horizontal lines signify the 5% significance levels. The time evolution of the path coefficients shows weakening and breakdown of Z1🡪Z2 and Z2🡪Z4 causal links of EWP and IVTW🡪VIMDI, SSTP🡪IVTW, VIMDI🡪PR links of TMP during the months of DJF.

Fig. S11: Evolution of the ENSO driven EWP and TMP causal pathways tested under PCMCI+ from November to March. For this analysis we imposed in all months the November precursor regions (location and shape) and extracted the corresponding precursor time series for each observable and month. The EWP and TMP causal pathways of ENSO to HMA PR break down during December to March with broken links (Z1🡪Z2, SSTP🡪IVTW, VIMDI🡪PR) and altered directions (Z4🡨Z5). The PCMCI+ testing was performed at τ_max_=2, and 5% significance level.

Existing literature suggests that ENSO has an active teleconnection footprint in the tropical and north Atlantic in November having causal pathways directed towards North Atlantic-European (NAE) region, which vanishes in subsequent winter months (Sabatani and Gualdi, 2025). Specifically, the Niño3.4 teleconnection dominates in November, whereas in December, the Tropical West-East Indian Ocean (TWEIO) teleconnection prevails, reinforcing the positive North Atlantic Oscillation (NAO). Further insights from studies reveal that the ENSO can also cause phase reversal of NAO during December-February, i.e. El Niño triggering a negative phase of NAO while La Niña causing a positive NAO (Toniazzo et al., 2006; Jiménez-Esteve and Domeisen, 2018; Geng et al., 2023), which could lead to different teleconnection outcomes. Our findings show consistency with literature, i.e. prevailing ENSO teleconnection towards HMA PR during November and the possibility of other modes (such as NAO and TWEIO) breaking the ENSO pathway during the mid-winter period.

It is worth noting that we have also looked at physically relevant and persistently active MJO years to see if excluding those years from the analysis results in a significant correlation of ENSO with HMA precipitation, as this could imply that MJO is causing the disruption of the ENSO teleconnection pathways. For this, we identified years with active MJO, i.e. OMI magnitude ≥1 for at least 15 days in a month, and phases 2-4 and 6-8 during December-February respectively (phase 2-4 of MJO in the equatorial Indian ocean region has a suppressing effect in winter Asian precipitation above 25°N, while phase 6-8 MJO in the western equatorial Pacific tends to have an enhancing effect in the same region; *Anandh and Vissa, RMetS Meteorological Applications, 2020*). By excluding these years from the 70 years of record, we found no significant change (at the 5% level) in the ENSO-HMA PR correlations during DJF, indicating that while MJO subseasonal variability may have an impact over HMA precipitation, it does not seem to disrupt the ENSO-HMA causal pathways at the monthly scale. Further analysis at the weekly time scale might provide more insight on the MJO influence on HMA precipitation but falls outside the scope of this study, which focuses on ENSO-driven causal pathways to HMA PR.

**References:**

1. Anandh PC and Vissa NK (2020) On the linkage between extreme rainfall and the Madden–Julian Oscillation over the Indian region, RMetS Meteorological Applications, 27, e1901, <https://doi.org/10.1002/met.1901>.
2. Geng X, Noh KM, Kim K. et al. (2023) Midwinter breakdown of ENSO climate impacts in East Asia. npj Clim Atmos Sci **6**, 155, <https://doi.org/10.1038/s41612-023-00474-4>.
3. Jiménez-Esteve B and Domeisen D (2018), The Tropospheric Pathway of the ENSO–North Atlantic Teleconnection, 31, 11, <https://doi.org/10.1175/JCLI-D-17-0716.1>.
4. Pearl, J (2013) Linear models: A useful microscope for causal analysis, Journal of Causal Inference, vol. 1, no. 1, pp. 155-170. <https://doi.org/10.1515/jci-2013-0003>.
5. Sabatani D, Gualdi S (2025) ENSO teleconnections with the NAE sector during December in CMIP5/CMIP6 models: impacts of the atmospheric mean state, npj Climate and Atmospheric Science, 8, 226, <https://doi.org/10.1038/s41612-025-01064-2>.
6. Toniazzo T, and Scaife AA (2006) The influence of ENSO on winter North Atlantic climate, Geophy. Research Letters, 33, L24704, <https://doi.org/10.1029/2006GL027881>.
