# Supplementary material for: The three causal pathways of ENSO teleconnections to High Mountain Asia winter precipitation
Source: Clim Dyn. 2026 Jun 1;64(6):267. doi: 10.1007/s00382-026-08198-w (PMC13226391; doi:10.1007/s00382-026-08198-w)
Supplement: Supplementary file 2 — Supplementary file2 [file 382_2026_8198_MOESM2_ESM.docx]

**Supporting Data Links**

Precipitation: TSv4.08, UK-CRU, 1951-2020, monthly, 0.5^0^×0.5^0^

*(https://crudata.uea.ac.uk/cru/data/hrg/index.htm#current*)

Sea Surface Temperature: HAD-SST, UK-Met Office, 1951-2020, monthly, 1^0^×1^0^

*(https://www.metoffice.gov.uk/hadobs/hadisst/data/download.html*)

Atmospheric Variables: ERA5, 1951-2020, monthly, 0.5^0^×0.5^0^ at single and pressure levels

*(https://cds.climate.copernicus.eu/datasets*)
